# Metas-Chip precisely identifies presence of micrometastasis in live biopsy samples by label free approach

**DOI:** 10.1038/s41467-017-02184-x

**Published:** 2017-12-19

**Authors:** Mohammad Saeid Nikshoar, Mohammad Ali Khayamian, Saeid Ansaryan, Hassan Sanati, Milad Gharooni, Leila Farahmand, Farshad Rezakhanloo, Keivan Majidzadeh-A, Parisa Hoseinpour, Shahrzad Dadgari, Leila Kiani-M, Mohammad Saqafi, Masoumeh Gity, Mohammad Abdolahad

**Affiliations:** 1 Nano Bio Electronic Devices Lab, School of Electrical and Computer Engineering, College of Engineering, University of Tehran, P.O. Box 14395/515, Tehran, Iran; 2 Nano Electronic Center of Excellence, Thin Film and Nanoelectronic Lab, School of Electrical and Computer Engineering, College of Engineering, University of Tehran, P.O. Box 14395/515, Tehran, Iran; 3 Integrative Oncology Department, Breast Cancer Research Center, Motamed Cancer Institute, ACECR, P.O. Box 15179/64311, Tehran, Iran; 4 Genetics Department, Breast Cancer Research Center, Motamed Cancer Institute, ACECR, P.O. Box 15179/64311, Tehran, Iran; 5 Central lab of Pathobiology, Department of Pathology, Breast Cancer Research Center, Motamed Cancer Institute, ACECR, P.O. Box 15179/64311, Tehran, Iran; 6 Department of Radiology, Breast Cancer Research Center, Motamed Cancer Institute, ACECR, P.O. Box 15179/64311, Tehran, Iran; 7 Advanced Diagnostic and Interventional Radiology Research Center, Tehran University of Medical Sciences, P.O. Box 14155/6447, Tehran, Iran; 8 Department of Radiology, Medical Imaging Center, Tehran University of Medical Sciences, P.O. Box 14155/6447, Tehran, Iran

## Abstract

Detecting the micrometastasis is a major challenge in patients’ survival. The small volume of the biopsied tissue results in limited number of histopathological samples and might reduce the rate of accurate diagnosis even by molecular technologies. We introduce a microelectronic biochip (named Metas-Chip) to detect the micrometastasis in unprocessed liquid or solid samples. It works based on the tendency of malignant cells to track single human umbilical vein endothelial cell (HUVEC)-sensing traps. Such cells detach themselves from the biopsied sample and invade the sensing traps by inducing membrane retraction and blebbing, which result in sharp changes in electrical response of the sensing elements. Metas-Chip identified the metastasis in more than 70 breast cancer patients, in less than 5 h. Moreover it detected the metastasis in lymph nodes of nine patients whom were missed by conventional pathological procedure. Multilevel IHC and real-time polymerase chain reaction (RT-PCR) tests confirmed the diagnosis.

## Abstract (web summary)

Detecting metastatic cells in tumor/lymph node samples of breast cancer patients is extremely important for diagnosis. Here the authors develop a microelectronic biochip that detect the presence of invasive/metastatic cells in unprocessed biopsies and performs better than the current gold standards.

## Introduction

Metastasis happens when cancer cells acquire a migratory to invasive phenotype, initiated from groupings of cells that appear to break off from primary tumors^1,2^. Invasive phenotype of such cells is in correlation with their invasion to endothelial vascular layer in the beginning of the metastasis^3–6^. Identifying metastatic cancer cells in a sample resected from the secondary tissue of the patients by core needle biopsy (CNB), endoscopy, colonoscopy, and fine needle aspiration (FNA)^5^ is the most important step in cancer staging and therapeutic regimes. Existing pathological methods are designed to track the presence of abnormally aggressive cells in the fixed samples prepared from removed tissues by cytological^6,7^ and immunohistochemical staining procedures^8^. Although cancer cells are detectable in some cases, they might be rare or only exist in regions of the removed sample that are not investigated by the pathologist^9^, and preventing missing any aggressive cancer cells is time consuming and expensive. Here we developed a microchip technology (Metas-Chip) to detect the presence of invasive/metastatic cells in unprocessed tumor/lymph node samples of breast cancer patients. Metastatic cells actively detach themselves from the sample by their own invasive tendency to the biochemical signals released from single-HUVEC-sensing traps^10–12^, which have been positioned and cultured on gold microelectrodes by dielectrophoresis. Then, the trap is assaulted by metastatic cells and is retracted, and the electrical response exhibits more than 70% changes in less than 4 h. The results of Metas-Chip were compared by H&E reports of the patients and non-similar results were rechecked by multilevel IHC and RT-PCR assays^13,14^. This approach enables specific and label-free efficient capture of metastatic cells with a simple, fast, and chemistry-free method in small biopsy samples, which will improve the diagnostic impact of CNB and FNA before surgery or therapeutic treatments.

## Results

### Design of the Metas-Chip

**Fig. 1:**
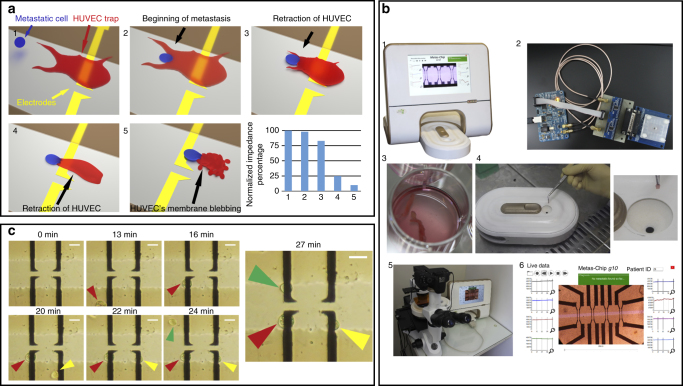
Design and operation of the Metas-Chip. **a** The system captures and diagnoses metastatic cells from the unprocessed CNB or FNA sample derived from the lymph of any suspicious secondary tissue, based on their active interaction by single vascular traps and retraction of the trap due to invasion of metastatic cell. This sharply reduces the electrical resistance of the sensing region. **b** Image of a working Metas-Chip with complete accessories (b1) including multiple rows of microelectrodes selectively covered by single HUVECs that form consecutive traps for metastatic cells, electrical interfacial boards and matching optical-electrical measurements subsystem (b2); sentinel lymph sample of a patient (b3) is held on the inlet without any processing (b4). High-magnification optical-capturing system takes live images from any interaction between single traps and probable detached cells from the sample (b5). b6 shows the software user interface (UI) of Metas-Chip, which include the microscopic image of sensing region with the micro-electrical traps collection networks in live matching by time-dependent electrical diagrams of all sensing traps. Occurrence of metastatic interaction would be recognized and presented by the system. The size of the main detecting chip is 0.5 × 0.5 cm^2^. **c** To prepare the chip for detection of metastasis, single HUVECs selectively covered each electrical trap by dielectrophoretic process (electrostatic driving force biased on the system). The scale bars are 25 µm in length

The Metas-Chip detects metastatic cells, in either solid or liquid biopsies, by relying on the strength of their invasion to retract single HUVEC from electrical sensing traps (Fig. 1a). The live biopsied samples are floated in a cavity embedded on top of the chip surface (Fig. 1b1–5) filled by dulbecco’s modified eagle’s medium (DMEM) media solution. A couple of electrodes selectively covered by a single vascular cell (by the assistance of electrostatic and dielectrophoretic cell patterning (Methods)) make up the basic unit of the chip. The couple electrode unit with the size of 10 and distance of less than 10 μm is repeated in multiple rows for redundancy (Fig. 1b6). So at least more than 15 metastatic cells could interact with one chip (include 15 single-HUVEC-sensing traps) at the same time. Each HUVEC trap would individually cover one sensing electrode, and if being retracted by a metastatic cell, a drastic change in electrical response of the electrode would be occurred. Presence of HUVEC-sensing traps stimulates the metastatic cells existing in the biopsied sample due to various suggested biological mechanisms^4^. Although many mechanisms were proposed on the attraction of invasive cells to endothelial barrier^3,15^, the precise reason behind this phenomena is still not clear. Many molecular functions and complicated signaling mechanisms were suggested to play a role in invasion of cancer cells to endothelial barrier^16^. Some reports stated that different enzymes produced by endothelial vascular cells attract metastatic cells and facilitate the formation of tumor-cell invadopodia^10–12^. Presence of matrix metallo proteinasse (MMP) proteins at the external sites of invadopodia are so crucial in their ability to proteolyse and disturb vascular cells^12^, which is distinguishable by retraction of HUVECs followed by their membrane blebbing (Supplementary Movie 1). Hence, invasion to the vascular endothelial barrier is one of the imminent steps of metastasis followed by entrance of cancer cells’ nano-conduits into HUVECs and disturbing their morphology and proliferation^15^. A cavity embedded on top of the chip surface, to locate the biopsied sample, funnels the detached cells through the single-sensing traps to mediate the invasion conditions. Metas-Chip is optimized to handle the active migration of the detached malignant cells without using any micro-pumps to induce flow speed. An invert microscopic imaging system (with phase contrast and fluorescent equipment) is embedded at the bottom of the chip substrate to record any invasive interactions in real-time. An analyzer software (written by C#) matches the electrical response of the traps with time-lapse images of the interactions to evaluate the syndicate between sharp reduction of electrical signals and HUVEC retraction by invasive/metastatic cells. Observing a sharp reduction (80%) in electrical diagrams of at least one sensing trap, 4 h after exposing the biopsied sample or metastatic cell lines to the chip, indicates the invasive interaction between a cell and a HUVEC trap (Fig. 1a). If this sample has been resected from the primary organ, the cancer is invasive and if it has been removed from the peripheral tissues such as sentinel lymph nodes (in breast cancer), the cancer has entered micrometastatic stage.

### Selective cell patterning to form single-HUVEC-sensing trap

We have applied an electrically active positioning system as a preferred cell-registration technique to safely and rapidly place single HUVECs on the sensing microelectrodes. Here, HUVECs are individually adhered to the microelectrodes using dielectrophoresis (DEP) (Supplementary Movie 2), the force on polarizable bodies in a non-uniform electric field^17,18^. These registered single cells play the role of active trap to capture the metastatic cells. HUVECs would maintain their positions after adhesion on the microelectrodes. The details of selective patterning procedure were discussed in the Methods. The entire array of single HUVECs patterned on the microelectrodes is formed within several minutes (Fig. 1c) and they would be spread to cover the surface of registered electrode in less than 5 h.

### Chip characterization and optimization using cell lines

To test the validation of the device, first we used an array of individual sensing traps and investigated the non-metastatic and metastatic cell lines from breast cancers (MCF7^19^ and MDAMB468^20,21^, respectively) in interaction with the Metas-Chip.

The electrical impedance of single-HUVEC-covered-sensing traps was measured as 4 kHz, the best frequency to be ensured of attachment of the cell^19,22^ by our designed multiplexed readout board as the background response. After complete spread of each single-HUVEC on the sensing trap, more than 80% of the electrical current flowed through the electrode would be blocked. So we define a global response for all of the sensing traps in Metas-Chip related to the percent of blocked current. This would be equal to the increased impedance of the electrode. When a HUVEC completely covers whole of an electrode, the impedance blocking in 4 kHz is about 100% and the response of the sensing trap was assumed as 1. In contrast, if the endothelial cell completely has been detached from its assigned single electrode, the impedance blocking would reach 0%, and hence the response is assumed to 0. Any metastatic/invasive interaction with HUVEC-sensing traps must retract them from the electrodes and reduce the response of the electrode to about 0.

**Fig. 2:**
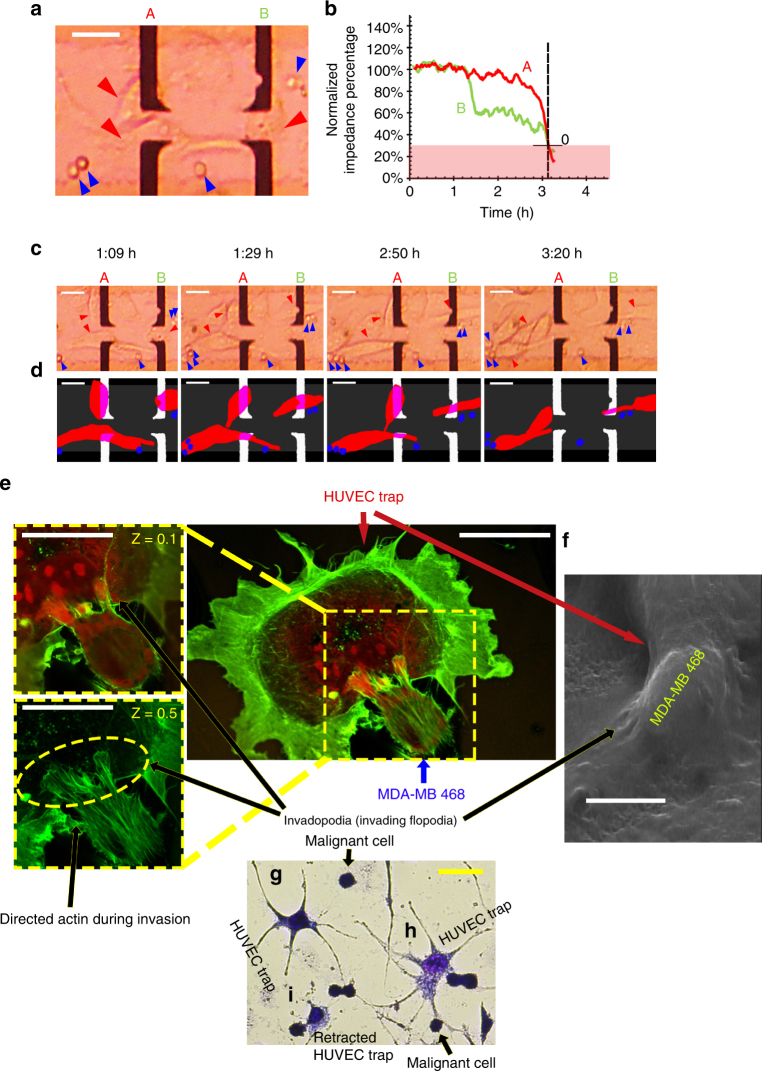
Interaction between sensing traps and malignant cell lines. **a** Optical image from two MDA-MB 468 (human breast metastatic carcinoma) clusters captured by the sensing traps of Metas-Chip. **b** Characterization of the Metas-Chip using MDA-MB 468 cell line spiked in whole solution. **c** Time-lapse optical images, which shows high correlation with schematic (**d**) explaining the dynamic balance responsible for the capture of metastatic cells and sharp reduction in the response occurs when the single vascular cell being detached from the sensing electrode by metastatic cells. Forces acting on the HUVEC traps just have been initiated from metastatic attraction of cancer cells as, no drag forces, no fluid flow and no reaction forces applied from the system. Individual cells are <10 μm wide, and the sensing electrodes covered by single HUVECs are >15 μm wide. Whole metastatic interaction detected by the system has been occurred in less than 3.5 h. **e** Confocal images from invasion of MDAMB468 cell into single-HUVEC trap. Directional assembly of the actin microfilaments and formation of invadopodia entered into the membrane of vascular cell are the results of receiving chemokines S100A8 and S100A9 chemical signals from the HUVEC by chemo-sensing part of the metastatic cells located in the external of filopodia and reinforced by MMP2 family. Various images taken from the cells in two different heights (*Z* = 0.1 and *Z* = 0.5) better presented the entrance of metastatic cell’s invadopodia into the HUVEC trap. **f** FE-SEM image of a vascular cell invaded by a metastatic breast cell also presented the direct interaction of caner cells' invadopodias with the HUVEC’s membrane. Geimsa stain image from the interaction between MDA-MB468 cells and HUVECs presented: **g** a non-invaded trap; **h** start of invasion; **i** invasion and start of retracting perturbation induced into HUVECs depend on the start time of each interaction. The scale bars are 25 µm in length

We introduced rare concentrations of MDA-MB 468 and MCF-7 cells separately into the cavity of individual chips (50 cells#/ml) (Fig. 2, Supplementary Figure 1, respectively). Only MDA-MB 468 cells invaded HUVEC traps which retracted them from the surface of the electrodes in about 4.5 h and lowered the electrical response of the single-sensing trap to 0.2 (Fig. 2a–d). Live matching (between electrical response and optical image) system of Metas-Chip would elaborate the time correlation between the metastasis-induced HUVEC retraction and ~75% reduction in electrical response of the traps (*P* < 0.05 with respect to non-retracted sensing trap calculated by Chi-squared method). In contrast, MCF-7 cells did not present any aggressive interactions by HUVEC-sensing traps (Supplementary Figure 1a,d), and no changes in the electrical response was observed. Even, the presence of a single metastatic MDA-MB468 cell could induce electrical spike response in a sensing trap. This might be correlated with a strong tendency of metastatic cells to invade the HUVEC layer.

After 5 h from the introduction of the cell solution to the individual devices, the Metas-Chip captured 4/5 MDA-MB-468 cells and 0/200 of MCF7 cells in a great match between HUVEC retraction from the electrodes (recorded by microscope) and significant reduction in the electrical response (measured by readout unit). We used confocal immunofluorescent assay to evaluate the mechanism of invasion during extraction of the vascular traps (Fig. 2e). Bundled filopodias (named invadopodia), formed during the extravasation of metastatic cells^10^, could be observed in external membrane of MDA-MB468 cell as sharply colored niches. Z (height) scanning of the cells by confocal imaging also indicated the entrance of the metastatic cell into the HUVEC. Field emission scanning electron microscopy (FE-SEM) taken from a vascular cell during being invaded by the MDA-MB468 cell also indicated the formation of invadopodia in cancer cells (Fig. 2f). No trace of invadopodia formation, actin remodeling, and invasive attachment were observed in confocal (Supplementary Figure 1e) and FE-SEM (Supplementary Figure 1f) images of MCF-7 cells after being interacted by HUVEC traps. Moreover, Giemsa cytopathological images also showed the HUVECs being invaded by various MDA-MB 468 cells depending on the start time of each interaction (Fig. 2g–i).

Such investigations would ensure us about the possibility of cell post analysis to drive further biological data on the metastatic/invasive nature of captured cells from the lymph/tumor sample.

### Identification of invasive cells in biopsied samples

We applied Metas-Chip to CNB samples collected by interventional radiologist from the breast tumor and lymph nodes of 40 patients (36 females and 4 males) with breast cancers. Additional sample study were done on surgically removed samples of some patients (2/40). Moreover, 30 samples resected by FNA from additional patients, which contain much less cellular concentration than CNB, were also investigated by Metas-Chip. Minor part of each sample was tested by Metas-Chip (Fig. 1 b4), and the major part (Fig. 1 b3) was prepared in parallel for pathological assays which includes H&E, IHC, and RT-PCR (Methods). After 4–5 h of live recording the optical and electrical data, presence of metastatic/ invasive cells in the lymph/ tumor sample could be achievable by the Metas-Chip analyzing software package (Fig. 1 b6).

The Metas-Chip diagnostic principle is fundamentally different from that of marker-based pathological methods. It captures metastatic cells in a freshly removed solid or liquid sample in single or cluster forms due to their invasive activity regardless of their morphology and marker-binding affinity, thus allowing detection of metastatic cells that might otherwise escape from labeling and staining. Moreover, captured cells are retained under a live dynamic function, unlike with assays where cells are fixed, lysed, or exposed to damaging stresses (IHC, RT-PCR,…)^23,24^. So they could be reanalyzed by marker-based methods such as epifluorescence microscopy. Finally, other types of the cells existed in the biopsied tissue like noninvasive epithelial cells, peripheral lipids, and blood cells don’t apply invasive interaction by HUVEC traps, so these types of cells wouldn’t be captured by the Metas-Chip.

**Fig. 3:**
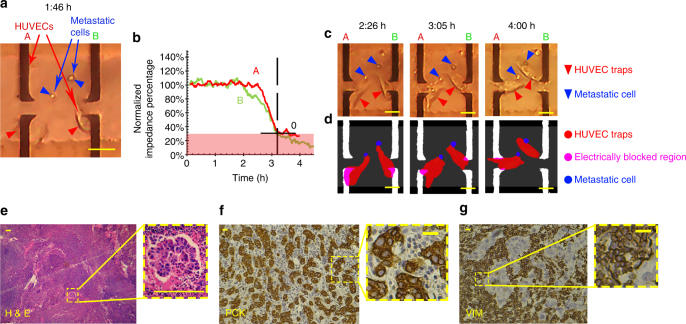
Capture of metastatic cells in SLN samples of a patient with metastatic cancer by both Metas-Chip and pathological process. **a** Representative images of two live metastatic cells detached themselves from the isolated lymph of the patient with metastatic breast cancer and attacked two individual single-HUVEC-sensing traps. **b** Their invasion induced sharp reduction in electrical response of the traps by retracting the HUVECs from the sensing electrodes, which could be traced in time-lapse optical images (**c**) and simultaneous schematics (**d**). H&E and immunohistochemical characterization of the patient’s SLN: (**e**) Images of a lymph region stained with hematoxciline and eosine. Nest of tumor cells with hyperchromic nucleus could be observed. This would indicate that the lymph node has been metastasized by invasive ductal breast carcinoma. (**f**) Pan cytokeratins and (**g**) vimentin expression in the sentinel lymph node of the patient with metastatic breast carcinoma. High reactivity of the cells with the markers (brown-yellow faint colors in the images) indicates the sharp involvement of the lymph with tumor cells. The scale bars for **a**, **c**, **d**, **f** and **g** are 25 µm in length and the scale bar for **e** is 100 μm in length

Metas-Chip scored as positive those samples that (i) induced at least one reductive spike (1 → 0) in the electrical response diagram of at least one single-HUVEC-sensing trap. (ii) The metastatic interaction was lively observed in the time-lapse imaging system of Metas-Chip. (iii) The electrical responses and optical images exhibited great syndication. A representative live metastatic cell, detached itself from the biopsied SLN sample of a patient (with metastatic breast cancer: ID1) is shown during invasion to a HUVEC-sensing trap and subsequent retracting it from the electrodes in less than 4.5 h (Fig. 3). As a result, Metas-Chip reported a considerable reduction in electrical response of two invaded traps (Fig. 3a, b). This matching could be observed in simultaneous images derived from the optical captures (Fig. 3c). It is worth noting that the time-lapse images of all the individual traps exhibited reductive spikes, were captured during the metastatic interaction (Fig. 3d). So, the patient was scored as positive for metastasis.

The standard diagnostic methods applied on the samples to be compared with the Metas-Chip was hematoxylin–eosin (H&E) staining^6^. H&E has been used by pathologists for about a hundred years. Hematoxylin stains cell nuclei blue, while eosin stains cytoplasm and connective tissue pink. Due to the long history of H&E, well-established methods, and a tremendous amount of data and publications, there is a strong belief among many pathologists that H&E will continue to be the common practice in future^25^. As could be observed in the H&E images of the sentinel lymph nodes of the patient (Fig. 3e) the nest of tumoral cells with hyperchromic nuclei is distinguishable in lymph node structure. Such result corroborated the correct detection of Metas-Chip in this patient which were confined by advanced immune marker-based imaging (Fig. 3f, g). Also, in the patients diagnosed as negative lymph nodes by Metas-Chip (ID: 29), in which none of the detached cells from the biopsy samples invaded to the single-HUVEC traps (Supplementary Figure 2a–d), H&E results indicated reactive lymphoid hyperplasia without any signs of malignancy (Supplementary Figure 2e).

All of the lymph node samples biopsied from the patients with metastatic cancers based on the H&E diagnosis were shown with on-chip capture of metastatic cells by Metas-Chip (Table 1 & Supplementary Table 1). Moreover, all of the biopsied breast tumors diagnosed as invasive carcinoma in H&E, exhibited invasion to at least one sensing trap in Metas-Chip (Supplementary Tables 2 & 3).

**Table 1:** Metas-Chip, H&E, IHC and RT-PCR diagnostic results of breast lymph nodes removed from breast cancer patients by CNB

Expression of Vimentin(Vim) and Pancytocheratin(PCK) markers were assayed by IHC as a reference diagnosis in CNB samples. Detection of metastasis in each assay is correlated with expression levels of transcripts associated with the presence of malignancy in the lymph region such as Vimentin, N-Cadherin, MMP2, and MMP9. The trace of transcripts in suspicious lymph nodes are sharply distinguishable than safe samples which indicates the accuracy of Metas-Chip

**Fig. 4:**
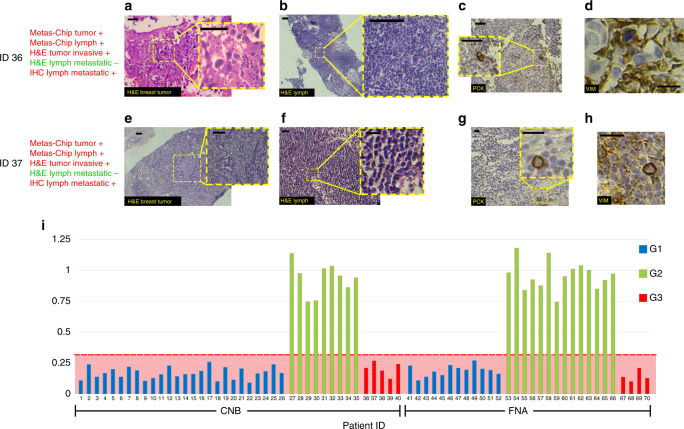
H&E stained from breast specimen of patients present invasive ductal carcinoma. **a**, **e** Meanwhile H&E staining results from the lymph nodes of the patients presented no trace of tumor cells (**b**, **f**). Multilevel IHC staining from the lymph nodes of the patents revealed the presence of at least one tumor cell sharply expressing the PCK (**c**, **g**) or Vimentin (**d**, **h**) for both suspicious patients. **i** Diagnostic chart of Metas-Chip showing “final electrical response of the fourth hour after adding the lymph sample to Metas-chip/Initial electrical response before adding the sample” for all patients. The scale bars are 25 µm in length

Metas-Chip identified the presence of invasive cells in the biopsied tumor samples of 20 patients with invasive breast cancer diagnosed by H&E and Pap staining (16 CNB and 4 FNA samples). Also, it tracked the metastasis in all 38 lymph nodes diagnosed as metastatic breast cancer by H&E and Pap staining (26 CNB and 12 FNA samples). But, it captured metastatic cells in the lymph node samples of 9/70 patients (5 CNB and 4 FNA samples) (Supplementary Figure 3a–d: patient ID36 & Supplementary Figure 3e-h: patient ID37) diagnosed as non-metastatic lymph node by H&E staining methods (Fig. 4a, b: patient ID36). Hence, these patients were assumed as doubtful people to metastatic cancer. In this regard, we categorized the patients in three groups due to the results of their lymph node assay by conventional pathological staining method (H&E, Pap Staining) and Metas-Chip; G1: known metastatic cases (positively scored by both conventional pathological staininig and Metas-Chip), G2: known safe lymph nodes (negatively scored by both conventional pathological staining and Metas-Chip), and G3: doubtful cases (negatively scored by conventional pathological staining, but positively scored by Metas-Chip). No patient was observed with negative score in Metas-Chip, but positive score at same time in conventional pathological staining.

### Immunohistochemical and molecular analysis of patients

To test the versatility of the Metas-Chip for accurate addressing the presence of metastatic cells in core needle-biopsied SLNs, (specially in patients from G3), we applied immunohistochemical markers of metastatic cells on the samples through IHC assay as a more advanced staining technique, which makes use of antibodies to highlight specific antigens in the tissue^8^. IHC can be employed to investigate the earliest changes in transformed tissues, identifying metastatic-associated cellular changes might not normally visible with H&E.

A range of the most recognized metastatic-associated proteins were investigated on the lymph samples of the patients selected from all three groups. IHC standard reports indicated the expression of PCK^26^, Vimentin^27–29^, MMP2 and MMP9^30^, and overexpression of N-Cadherin 1^31^ in SLNs of breast cancer patients with metastasis. Some of these markers such as Vimentin and MMP2&9 are accurate indicators for the invasive nature of the found cancer cells. To be ensured from the micrometastasis, presence of the cells expressing PCK and Vimentin were deeply investigated by preparing multilevel IHC from the SLNs of the patients from all groups with special consideration on G3 (doubtful patients).

Figure 3f, g, Supplementary Figure 2f, g, and Figure 4c, d present the IHC images on the expression of cancer-associated markers (PCK and Vim) in some SLNs samples from G1, G2, and G3 patients, respectively. Expression of PCK (Fig. 3f) and Vimentin (Fig. 3g) in SLNs of the known metastatic cases (G1) confirmed the correlated diagnosis of both Metas-Chip and H&E for those patients (Table 1 patients ID 1–26 & Supplementary Table 1 patients ID 41–52). Also, negative expression of PCK (Supplementary Figure 2f) and Vimentin (Supplementary Figure 2g) in the SLNs of the known non-metastatic cases (G2) supported their safe lymph nodes as indicated by both Metas-Chip and H&E (Table 1 patients ID 27–35 & Supplementary Table 1 patients ID 53–66). To present a complete set of data for better tracking, diagnostic results achieved from the breast tumor samples biopsied from some of the patients are presented in Supplementary Tables 2 and 3. It is observable that all of the tumors exhibited invasive carcinoma and could be candidate cases for investigating the metastasis in their lymph nodes.

**Fig. 5:**
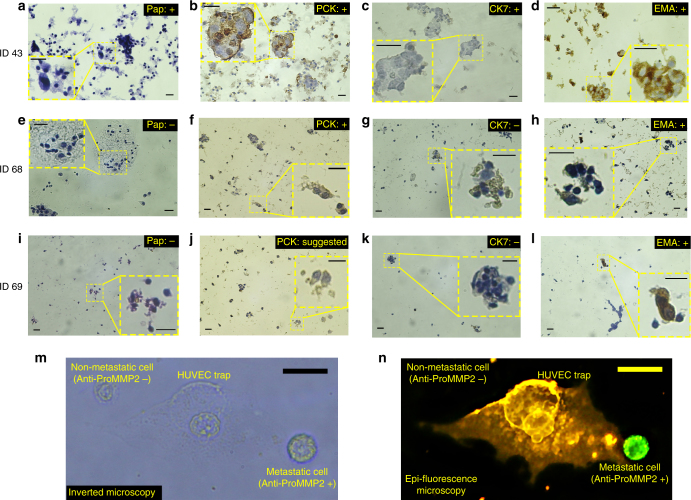
Cytopathological and immunohistochemical images of the lymph node aspirated from a known metastatic sample (ID 43) positively scored by Metas-Chip in comparison with the similar assays from two suspicious aspirated samples (ID 68 and 69) negatively scored in Pap stain, but positively scored by Metas-Chip and IHC. **a** Cancer cells with large hyperchromic nucleus present metastatic carcinoma, meanwhile no trace of malignant cells could be observed in (**e**, **i**). Expression of PCK in the cancer cells are observable in metastatic (**b**) and one of the suspicious patients (**f**). Also the expression of PCK is suggestable in lymph of other suspicious patient (**j**). Expression of CK7 was positive in patient ID 43 (**c**) while it is negative in patient ID68 (**g**) and ID69 (**k**). Positive expression of EMA is observed in known metastatic (**d**) and suspicious patients (**h**, **l**). At least one IHC marker was positive in the suspicious patients who had been positively scored by Metas-Chip. Expression of metastatic epifluorescent marker (anti-proMMP2) on malignant cells invaded HUVEC trap. **m** Optical microscopy and (**n**) epifluorescence image of live single-HUVEC trap (stained by Dil: yellow) and breast cancer cells tagged for MMP2 (by anti-proMMP2: green) proteins presented in the surface of metastatic cells. Both of the cancer cells could be observed in optical images, but just one of them expressed the marker as active metastatic cells could be traced in epifluorescent image. Epifluorescent images of metastatic cell (expressed the marker) 90 min after invading the individual HUVEC trap showed entrance of the metastatic cell to vascular barrier . The scale bars are 25 µm in length

It is worth noting that we identified the trace of tumor cells expressed PCK and Vimentin in lymph nodes of all G3 CNB samples (Fig. 4c, d: patient ID36). Such results revealed the presence of micrometastasis in doubtful patients, which had been diagnosed by Metas-Chip. (e.g. Fig. 4e–h: ID37 & Supplementary Figure 4: ID38). Other groups reported that the breast cancer cells in epithelial to mesenchymal transition, would express both the PCK and Vimentin simultaneously, which might be the first signature of micrometastasis^32^. Most micro-metastasized breast cancer cells might appear to exist in a hybrid epithelial-mesenchymal state, a phenotype observed in instances of breast circulating tumor cells and consistent with the possibility of trans-endothelial migration^33^. Figure  4i presents a chart based on the diagnostic responses of Metas-Chip on the lymph node of all patients. Similar grouping (G1, G2, and G3) were corroborated by pap staining followed by deep EMA, PCK, and CK7 IHC investigations in FNA samples. These are popular markers for tracing the LN involvement in breast cancer patients resected by FNA. All of the FNA samples positively scored by Pap staining, had been diagnosed as involved ALNs in Metas-Chip (Fig. 5a–d & Supplementary Table 1: patients ID 41–52). Each suspicious FNA sample that negatively scored in cytopathology (pap staining), but positively scored by Metas-Chip, expressed at least one of the EMA, PCK, and CK7 markers (e.g. Fig. 5e–h: patient ID68 & 5i-l: patient ID69) (Supplementary Table 1).

Other interesting point was that following one of the doubtful patients (patient ID: 38), after the surgical resection of the lymph node (due to the surgeon’s opinion), indicated the involvement of 1/10 lymph’s frozen sections to malignant cells in H&E image. This is a hopeful achievement that detects a metastatic case from his/her lymph CNB sample before any pathological analysis would be possible by Metas-Chip.

Similar result had happened for one of the followed FNA-removed patient (patient ID 68).

Time-lapse imaging from the invasion process reveals that all of the metastatic cells migrate straight forward through the HUVEC traps and do not attach on the substrate of the chip before (Supplementary Movie 3). This corroborates the selective attachment of malignant cells to sensing traps with the assistance of their invadopodia (Fig. 2e) based on MMP receptors produced on their external region^21^.

Individual RNA sequencing analysis was performed to evaluate the expression of invasive/metastatic genes especially in lymph regions of the patients (Methods). Reference lymph and breast samples were prepared from a healthy donator.

The RT-PCR tests were investigated (Methods) on the patients from all of the three groups. Analysis of the results (Table 1) showed that: (i) All of the lymph nodes extracted from the G1 patients, expressed detectable levels of transcripts encoding metastatic-associated proteins (such as Vim, MMP2&9, and N-Cadherin) in comparison with a normal lymph sample; (ii) The primary breast tumor assayed in some of these patients (G1) expressed high levels of N-Cadherin; (iii) Almost all of the patients in G2 did not express Vim. Moreover, very-low expression of N-Cadherin and MMP9 as well as low expression of MMP2 were observed in the lymph nodes of all G2 samples. Significant expression of N-Cadherin in tumor samples assayed in patients from G2, revealed the invasive state of the breast tumors similar to G1; and (iv) All the patients from G3 (doubtful cases) expressed detectable levels of Vim as well as meaningful levels of MMP2&9. Expression of N-Cadherin was significant in two samples (Patient ID 37 & 39), meanwhile it was minor in the others. Trace of N-Cadherin was significant in the breast tumor samples of the patients from G3. The mentioned results of RT-PCR in doubtful patients indicated the trace of metastasis in their lymph nodes as another support to the precise diagnosis of Metas-Chip.

The impact of Metas-Chip in detecting the metastasis in the samples that could be hardly diagnosed even by IHC and RT-PCR is observable. It successfully assayed 70 samples resected by either CNB or FNA from the SLN and ALN of the individual patients and detected the invasive cancer cells more precise than conventional pathological staininig in a great demand with IHC and RT-PCR results. The low shear stress of the metastatic cells and biochemical signals received from vascular cells, facilitate the detachment of metastatic cells from original biopsied tissue to invade the sensing traps of Metas-Chip in less than 5 h (Supplementary Movie 4).

### Epifluorescent imaging of metastasis by Anti-proMMP2 marker

To more elaborate the metastatic nature of invaded cells captured by sensing trap, epifluorescent microscopy images were taken from the surface of the chip during the interaction with cancer cells based on Anti-MMP2 fluorescent marker (Abcam Co.). Presence of MMP family are so crucial in metastatic ability of cancer cells because they are secreted on external sites of invadopodia to facilitate proteolysis of endothelial cells targeted by a metastatic cell. The HUVEC traps on the Metas-Chip were stained by Dil (Abcam Co.) before interaction by the cancer cells. During the invasion of cancer cells, the surface of the chip was stained by the Anti-MMP2 epifluorescent markers (Methods). This marker would selectively bind to the MMPs presented on the surface filopodia of metastatic cells. Figure 5 m & n present the optical microscopy and fluorescent images of a HUVEC-sensing trap during invasion of metastatic cells respectively. Anti-MMP2 markers were strongly expressed on one of the cells (green colored in Fig. 5n), which revealed its metastatic nature. In contrast, no trace of the markers was observed on the other cell (top left in Fig. 5m), which did not apply any invasive interaction by the trap. Such results indicate the perquisite of bundled invadopodia with expression of MMP metastatic markers (Supplementary Figure 5) in a malignant cell capable to invade endothelial barrier. Moreover, high resolution confocal imaging from an invaded trap by a metastatic cell (in a FNA sample) revealed the alignment of its invadopodia, containing overexpressed MMP2 proteins, which resulted in destruction of the endothelial cell (Supplementary Figure 6 and Fig. 2e). Prognostic IHC markers such as Ki67 might not be reliable indications in metastatic diagnosis (Supplementary Figure 7) with respect to presented IHC and fluorescent markers.

### Interaction of immune cells (macrophages, lymphocytes etc.) by sensing traps

**Fig. 6:**
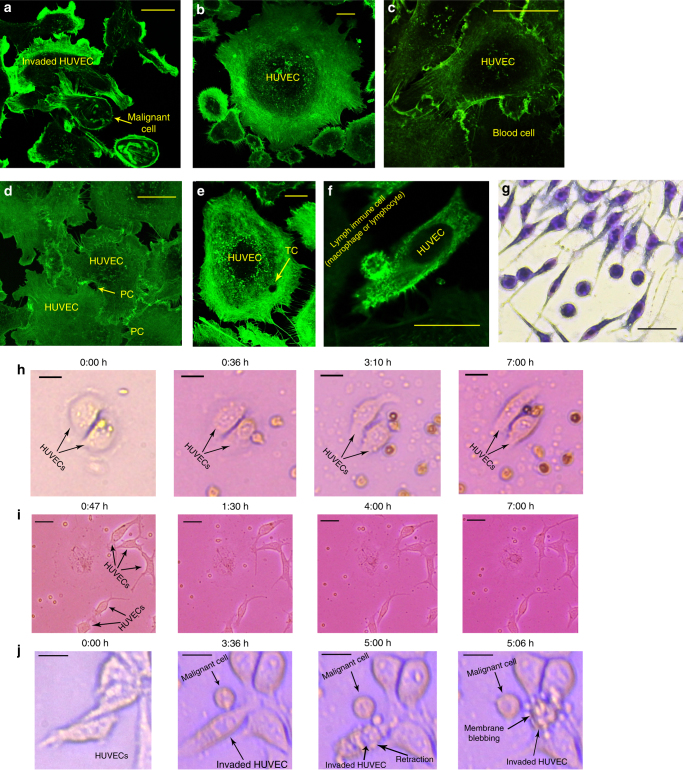
**a** Confocal images from the interaction of two individual malignant cell samples by HUVEC traps. Retraction of the membrane and signs of membrane blebbing could be observed. **b** Similar images were taken from the interaction of non-malignant lymph nodes and HUVECs, and no retraction was observed in the structure of interacted HUVECs. **c** Migration and diffusion of a non-malignant blood cell, derived from a healthy donator, into the HUVEC trap. **d** The trace of the hole produced by transcellular migration of leukocytes could be observed. **e** Similar confocal images from non-retracted endothelial layer after TC of blood immune cells . **f** Attachment of an immunocell existed in a non-malignant lymph node to the HUVEC. Note: all of the images were taken after at least 6 h of interaction between the cells and HUVECs. **g** Geimsa staining image taken after 7 h of interaction between WBCs (of a healthy donator) and HUVECs. Optical time-lapse images of **h** non-metastatic lymph; **i** healthy blood; and **j** metastatic lymph samples in interaction with single HUVECs. HUVEC retraction and membrane blebbing was only induced by the metastatic cell (**i**). Related Supplementary Movies: **g** Supplementary Movie 6 **h** Supplementary Movie 7 **i** Supplementary Movie 8. The scale bars are 25 µm in length

Metas-Chip did not record any invasive interaction between all of non-malignant lymph nodes and the sensing traps. It is known that, macrophages and some type of lymphocyte would intra/extravsate into/from the endothelial vascular barrier by paracellular (PC) and transcellular (TC) transmigrations^34,35^, but as their migration is a programmed pathway for endothelial cells^35^, they would not induce membrane blebbing (Fig. 6a) or retraction to the HUVEC barrier such as that observed in malignant invasion. The confocal images taken from the interaction of non-malignant lymph nodes (which contain various types of leukocytes^36^) and endothelial cells, presented in Fig. 6b–f, showed no perturbation induced on the HUVECs (Fig. 6b, c). The trace of PC (Fig. 6d), TC (Fig. 6e), transmigrations, and immunocell attachments (Fig. 6f), could be observed in non-retracted HUVECs after 7 h of interaction. This was also confined by Geimsa staining images taken from interaction between WBCs and HUVECs (Fig. 6g). Time-lapse images from the interactions between HUVECs and non-malignant lymph nodes presented neither retraction nor membrane blebbing of the HUVECs (Fig. 6h, Supplementary Movie 5). Similar interaction was observed for WBCs derived from the blood of a healthy donator (Fig. 6i, Supplementary Movie 6). In contrast, retraction and membrane blebbing were obvious in the HUVECs invaded by malignant cells (Fig. 6j, Supplementary Movie 7). It was observed that although an immunocell, found in the lymph samples of a malignant patient, identified and tracked a metastatic cell, it could not stop it’s metastasis (Supplementary Movie 8, patient ID 43). Active functions of immune cells for identifying cancerous cells, bacteria, and any sign of inflammation would not induce any disturbance on endothelial cells (Supplementary Movie 9). As a result, any invasive retraction or membrane blebbing of HUVEC traps by non-cancerous cells were excluded. This technology enables scan-free processing of the whole-sample.

## Discussion

We introduced a bioelectronic technology that detects the metastasis in an unprocessed lymph node samples of cancer patients removed by core needle biopsy or fine needle aspiration. The dynamic capture of live malignant cells without any markers or labels offers important capabilities that are not readily achieved with current detection strategies. Invasion of metastatic cells that detached themselves from the sample through tracking the chemokine enzymes, secreted from the selectively spread HUVECs on the sensing microelectrodes, would retract the vascular cell. This results in an increased electrical current penetration through invaded single trap and informs us about the presence of metastatic cell in the sample. An integrated matched optical–electrical response monitoring system in Metas-Chip system tracks the trap (traps) invaded by metastatic cells. Existing technologies primarily target invasive cells in a part of removed samples in frozen state by H&E and IHC staining exhibit lower sensitivity and specificity for capturing rare metastatic cells, which might result in non-precise diagnosis and reduce the survival rate of the patients. Advanced immune purification assays involve multiple processing steps and would be so expensive and hard to be run on whole of the samples for all of the patients^8^. Moreover, expert operators and pathologists for staining and diagnosis are required, respectively. RT-PCR of metastatic-associated genes would be efficient for diagnosis of the malignancy, especially at the primary stages, but the low purity, especially in CNB-removed samples complicates downstream molecular analysis^13,14^.

Our epifluorescent, IHC and RNA sequencing data revealed the invasive nature of cancer cells. The fact that tracking the presence of such cells in the secondary tissue (such as sentinel or auxiliary lymph nodes) without any signs of histopathologically observable micrometastasis by this method, might be an early detection for malignancy, which has implications in clinical trials.

The results affirm that the reliance on active biological and electrical functions rather than on specific cancer cell surface epitopes to track metastatic cells in a non-processed solid or liquid sample, makes the Metas-Chip uniquely suited for investigation of metastasis. As limited number of patients were analyzed to validate these results, further studies will be required to support the use of this system in cancer diagnosis, which is under progress by our group.

As the Metas-Chip analyzes the samples in their vital state, some observations such as starting the analysis maximum 3 h after removing the biopsy samples from the patients or maintaining the temperatire of the sample reservoir before introducing to Metas-Chip in 37 °C ought to be respected.

Finally, some concerns such as quantitative grading of primary tumors and the threshold from micrometastasis to macrometastasis could be quantified based on the number and time interval of electrical spikes in sensing traps, which are our future trends to enhance the efficiency of Metas-Chip.

## Methods

### Metas-Chip fabrication and component design

Metas-Chip system comprises four main subsystem including core bioelectrical sensing traps, optical recording system, handling and incubating subsystem interracial electronic board, and matching-processing software. The devices were fabricated using combined photolithography and coating procedures. For mold fabrication, SU-8 photoresist (MicroChem) spun on a silicon wafer was patterned in the form of microfluidic channels through a chrome photomask by conventional photolithography. Poly(dimethylsiloxane) (PDMS) prepolymer and cross-linker (Sylgard 184, Dow Corning) mixed at 10:1 ratio was poured on the mold, first degassed and then cured at 65 °C for at least 4 h. The final device was built by bonding the cured PDMS peeled off from the mold and a glass substrate after surface activation in oxygen plasma. The fabricated devices were primed by flushing ethanol through microfluidic channels, and then washed using deionized water and PBS before use.

The Metas-Chip is composed of 16 single-parallel tracks, each equipped with single consecutive metastatic cell sensing traps, comprising a single HUVEC selectively covered whole of the electrode by DEP patterning. The biopsied sample is caught in little species and floated on top of these sensing traps that are designed to be on(1)/off(0) electrical switches through invasion of metastatic cells might be existed in the samples. The depth of the chip is 100 μm and the volume of the cavity including reservoir is about 200 μl.

### Metas-Chip software design and on-chip analytical procedure

Metas-Chip software is written in C# language using microsoft visual studio. The software records electrical signal captured by readout board and images taken by optical microscope camera and processes them. If an electrical signal is dropped below threshold (30%), the system warns the user about potential metastasis occurrence and shows the affected trap on screen for visual confirmation.

### Metas-Chip characterization and optimization

To characterize the capture sensitivity of the Metas-Chip, we used an analytical version of the Chip (with 16 single-sensing-trapping electrodes, in clinical model it has been increased to 80 uniformly distributed traps). The chip was covered by a mixed solution of 5 MDAMB468 and 50000 MCF10 breast cells. Checking the spiked traps in real-time by optical imaging systems revealed the invasive interaction of 4 metastatic cells with the traps (Fig. 2a–d). Non-captured MDA-MB-468 cell was traced by resuspending the solution on a surface and staining the sample by Anti-ProMMP2 marker, but no trace of any stained cell has been observed, which might be related to lower invasive ability of non-captured cells. As the main role of this system is detecting the presence of metastatic cell in peripheral tissues to diagnose probable micrometastasis, we did not test the system by higher concentration of metastatic cells.

### Characterization of metastatic cells before spiking

Fluorescently labeled MDA-MB-468 cells were prepared in singular and cluster architectures (see separate section on cell culture and reagents), and then a 2.5 μl cells suspension was deposited on an ultralow-attachment culture dish. The population was characterized by acquiring a fluorescence microscope image. The suspension was then re-pipetted, spiked into mcg 10 solution samples, and processed using the Metas-Chip at 37 °C supplied by incubating sub-system of the Metas-Chip . The culture dish was reimaged to account for metastatic cells that possible remain attached to the surface. The captured microscope images were post-processed, and cells within the testing sample were counted.

### Patients sample collection

Patients provided consent according to an approved protocol at breast cancer research center (Moatamed Cancer Institute, ACECR). Live spices from CNB samples were cut in similar specimens and directly transferred through the cavity of Metas-Chip immediately without any pre-processing. The test was separately done on the lymph samples (and some of the tumors) of each patient on individual chips. In the case of FNA samples, the sample already consists of suspended cells. The RBCs were removed from the sample with the assistance of Ficol followed by 20 min of centrifuge at 2000 RPMs. The RBC-removed sample was directly transferred to reservoir without any further processing.

After 4–5 h of live recording of the optical and electrical data, the presence of metastatic/invasive cells in the lymph/tumor sample could be achievable by the Metas-Chip analyzing software package (Fig. 1b6).

After testing the sample, the chip was exposed by trypsin to detach all of the adhered cells, and subsequently washed by alcohol, PBS, and DI water, to remove the non-specifically bound cells and debris. After measuring the electrical response of the cell-free electrodes. The HUVECs were again patterned on the chip and it was prepared for next test.

### Cell cultures and reagents

MCF7 and MDA-MB231 cell lines, were isolated from grades I and IV of human breast tumors, respectively. These cells were obtained from the standard cell banks of the National cell bank of (NCBI) located in the Pasteur institute and they were maintained at 37 °C (5% CO_2_, 95% air) in DMEM medium (Gibco) supplemented with 5% fetal bovine serum (Gibco), and 1% penicillin/streptomycin (Gibco). The fresh medium was replaced every other day. Human umbilical vein endothelial cells (HUVECs, ScienCell) were cultured in EC basal medium (EBM; Scien Cell) with additional 10% FBS, and guaranteed to sub-cultured for three population doublings.

All cell lines were tested and found negative for *Mycoplasma* contamination using conventional DAPI staining followed by fluorescent imaging. The cells were detached from the plates by trypsin and counted by neobar laam.

### Immunofluorescence staining

HUVECs covered on Metas-Chip were exposed by Dil (Sigma 42364) with the stain concentration of 20 μM. After 25 min holding in incubator, they were washed by PBS for three times. Subsequently, the media solution was added to the chip, and it was exposed to the sample of a patient or metastatic cell lines. After metastatic interaction between cancer cells and HUVEC trap, the cells captured on the Metas-Chip were fixed with 4% paraformaldehyde (PFA) and washed with PBS. Fixed cells were then permeabilized with 1% NP40 in PBS, blocked with 3% goat serum/2% bovine serum albumin (BSA), and immunostained with antibodies against MMP2 (abcam Ab37150) for breast cancer with the concentration of 2 µg/ml. The secondary antibody used was a goat anti-rabbit IgG conjugated to Alexa Fluor® 488 (green) (ab150077) used at a 1/1000 dilution. Stain-positive cells were detected using epifluorescent microscopy Optika Ltd. automated imaging system (Billerica).

### Characterization of actin filament assemblies in metastatic cells (during the invasion) by confocal microscopy

Actin microfilament distribution of metastatic cells during invasion to HUVEC trap were assessed by inverted confocal microscopy (Leica, TCS SP5, Germany). Prior to imaging, the cells were fixed in 4% formaldehyde for 15 min and permeabilized with Triton X-100 in PBS (with the concentration of 1%) for 5–10 min at room temperature. Then, all samples were washed and stained with the Actin-Phalloidin (Invitrogen A12379) (Green) and incubated for 30–45 min. The cell nuclei were stained with propidium iodide (PI) and (Invitrogen, USA). The Leica Application Suite Advanced Fluorescence (LAS AF) software (Leica Microsystems) was utilized to analyze the confocal microscopy pictures.

### DEP-based patterning of single HUVECs on sensing traps

The procedure for cell patterning based on DEP (Albrecht et al., 2006; Ho et al., 2006) was applied to obtain a selective and viable pattern of single HUVECs just on the sensing traps. First, HUVECs were suspended in the EGTA-containing DEP buffer and flown into the cavity of Metas-Chip. With the rate of 5 µl/min. Then, AC signal (5 Vpp, 5 MHz) was applied to the sensing electrodes to generate p-DEP forces to guide cells on the array of traps during the cell-seeding process, shaking the chip during cell patterning suppress from the physical attachment of the cells in non-desired places. Moreover, a calcium-containing DEP buffer without EGTA (0.75 mM of CaCl_2_; pH 7.0, 305 Osm, 2.74 × 10^−2^ S/m) could be injected at 5 µl/min to improve the detachment of non-patterned cells. Subsequently, we turned off the AC. After HUVECs were patterned singularly on sensing traps, DMEM with 10% FBS and 1% penicillin/streptomycin was injected at 5 µl/min to replace the calcium-containing DEP buffer. The time-lapse images taken from the patterning process by Metas-Chip optical system indicated the spread of the each HUVECs on a single traps in about 4 h. Finally, the cell-patterned electrodes was measured by the readout system to be ensured from the blocking of each sensing region by a HUVEC trap. Then the system is ready to be interacted by the biopsied samples of the patients.

As the cells are more polarizable than the surrounding media, the dipoles induced in the cells align parallel to the applied electric field^37^. The field is spatially non-uniform and the maximums would be occurred on the electrodes patterned on the surface of the chip. So, a resultant force due to DEP pulls the cells toward field maxima^37^.

When the polarity of the applied field is reversed, DEP continues to pull the cell toward the field maximum, allowing AC operation at high frequencies to reduce electrical loading of the cell membrane. After trapping a single HUVEC at the maximum region of the field, existing on the sensing region of microelectrodes, we flowed the media solution across the surface as a destabilizing force to remove the additional cells that might be trapped on non-desired places to implement the position of single HUVECs just on the sensing electrodes (Fig. 1c); the applied flows are powerful enough to remove the additional cells, but too weak to remove the strongly trapped cell directly above each electrode.

### Fine needle aspiration

Ultrasound-assisted FNA procedure: Initially the patients were informed about the reason for the procedure, the detail of the process, risks and benefits, and the existence of alternative techniques, and then they were asked to sign a term of free and informed consent approved by Tehran University of Medical Sciences International Review Board (TUMS IRB). Next, asepsis was performed in the axillary region, and about 3.5 ml of lidocaine at 2% was injected to the skin. The puncture was performed with a 21-gauge needle on a 10 ml syringe. In order to obtain the cytological material, the needle was moved in different directions (fan-shaped movements) maintaining vacuum that was unfinished before the removal of the needle. A sonographic image was acquired showing the tip of the needle within the target. Enough aspirates were obtained to use for both Metas-Chip and pathological slides preparation. The aspirates choosed for Metas-Chip were freshly injected to the DMEM solution, and the others were fixed with 95.6% ethanol, and later sent for cytological and immunohistochemical analyses.

### Procedure of progressive Papanicolaou staining method used for staining of FNA samples

This method applied to detect the presence of cancer cells in LNs of the patients removed by FNA, the nucleus is stained with hematoxylin to the intensity desired. The intensity of the nuclear staining is controlled by the immersion of the slide into a blueing agent. Most commonly used blueing agent is Sott’s tap water (pH 8.02). The staining protocol was presented step-by-step in Supplementary Table 4.

### Core needle biopsy

A CNB is much like previously conventional fine needle biopsy procedure. A slightly larger, hollow needle is used to withdraw small cylinders (or cores) of tissue from the abnormal area in the breast or sentinel lymph nodes. A CNB is done in the radiology part of the center with local anesthesia (you are awake, but your breast is numbed). The needle is put in 3–6 times to get the samples, or cores. This takes longer than an FNAB, but it’s more likely to give a clear result because more tissue is taken to be checked. A CNB can cause some bruising, but usually doesn’t leave scars inside or outside the breast.

The provider doing the CNB places the needle in the suspicious area using ultrasound to guide the needle into the right place. If the area is easily felt, the biopsy needle may be guided into the tumor while feeling (palpating) the lump. A small skin incision is made with a scalpel blade. The biopsy needle is then advanced manually under real-time US guidance. The outer cannula remains uncocked during manual needle advancement. In some cases (such as auxiliary lymph node biopsy), a tougher fascial layer under the superficial tissues necessitates a deeper incision or the use of a diamond-tipped guide cannula, and advancing the needle after cocking the outer cannula leaves a relatively thin and flexible portion of the stylet to withstand the insertion through the facial tissues, potentially compromising placement accuracy or even bending the needle at the collection trough, the thinnest portion. When the needle tip is just at or within the target, the gun is cocked once, which opens the trough without advancing the needle tip. After manually adjusting the position of the trough to center on the target, the operator releases the outer cutting cannula and closing the trough. The needle is inserted with the bevel facing up to facilitate penetration of the superficial tissues. Also the needle is advanced with the plastic flanges used to cock it optimally positioned so as not to compromise the angle of advancement. A portion of the trough bridge the cortex and surrounding fat, which helps the pathologist see the interface between the target tissue and surrounding normal (usually fatty) tissue, and thus more readily identify the target sample as being from a lymph node (Supplementary Figure 2).

The specification of the patients’ samples (BRISQ Summary/Checklist) is presented on Supplementary Table 5.

### Hematoxilin–eosin staining of the patients’ samples

Hematoxylin and eosin (H&E) staining is the most common staining technique in histopathology. This uses a combination of two dyes, hematoxylin and eosin used for demonstration of nucleus and cytoplasmic inclusions in clinical specimens. Alum acts as mordant, and hematoxylin containing alum stains the nucleus light blue. This turns red in the presence of acid, as differentiation is achieved by treating the tissue with acid solution. Bluing step converts the initial soluble red color within the nucleus to an insoluble blue color. The counterstaining is done by using eosin, which imparts pink color to the cytoplasm. The H&E staining process on a biopsied tissue starts with deparaffinizing the section, flaming the slide on burner, and placing it in the xylene. The treatment must be repeated. Then hydration must be done. The tissue section would be hydrated by passing through decreasing concentration of alcohol baths and water (100, 90, 80, 70%). In the next step, the sample is stained in hematoxylin for 3–5 min followed by washing in running tap water until sections “blue” for 5 min or less. The sample then is differentiated in 1% acid alcohol (1% HCl in 70% alcohol) for 5 min. Subsequently, we washed the sample in running tap water until the sections are again blue by dipping in an alkaline solution (eg., ammonia water) followed by tap water wash. Now, the sample is stained in 1% eosin Y for 10 min and washed in tap water for 1–5 min, and finally we dehydrate the sample in increasing concentration of alcohols and clear in xylene.

### Immunohistochemical staining of the patients’ samples

The study included 30 cases comprising of 20 retrospective and 12 prospective cases of sentinel lymph node-negative patients with breast cancer (from April 2002 to March 2007). A total of 178 mastectomies were performed on breast cancer during this time frame. Out of 178, 32 (17.97%) cases from G1 had positive sentinel lymph nodes on routine H&E staining, meanwhile cases from G2 and G3 had negative SLNs. All cases were selected for further IHC study. Two sections from each block from G1 and G2 samples and more than 8 sections from G3 samples were cut on poly l‑lysine coated slides. Antigen retrieval was performed by heat-induced epitope retrieval using microwave oven. IHC was performed using avidin biotin technique (using labeled streptavidin biotin (LSAB) + kit) with Dako Monoclonal anti‑Human PCK, clone AE1/AE3 (dilution 1:50), and Novocastra NCL‑Vim Human Monoclonal antibody (dilution 1:100). PCK staining gave brown cytoplasmic reactivity. Vim staining gave brown cytoplasmic reactivity with membrane enhancement. Cells were considered to be occult node metastases if they were Immunoreactive (expressed either PCK or Vim antigens) and found within the substance of lymph consistent with cancer cells^38^.

### RNA amplification and sequencing

RNA samples extracted from the biopsied tumor and lymph samples of the patients were thawed on ice and incubated at 70 °C for 90 s. To generate cDNA, we treated samples with reverse transcription master mix (0.05 μl RNase inhibitor, 0.07 μl T4 gene 32 protein, and 0.33 μl SuperScript III reverse transcriptase per 1 × volume) and incubated them on thermocycler at 50 °C for 30 min and 70 °C for 15 min. To remove free primers, we added 1.0 μl of EXOSAP mix to each sample, and then incubated the mixture at 37 °C for 30 min and inactivated at 80 °C for 25 min. Next, a 3′ poly(A) tail was added to the cDNA in each sample by incubating in master mix (0.6 μl 10 × PCR buffer II, 0.36 μl 25 mM MgCl_2_, 0.18 μl 100 mM dATP, 0.3 μl terminal transferase, 0.3 μl RNase H, and 4.26 μl H_2_O per 1 × volume) at 37 °C for 15 min and inactivated at 70 °C for 10 min. A second strand of cDNA was synthesized by dividing each sample into 4 and incubating in master mix (2.2 μl 10 × high-fidelity PCR buffer, 1.76 μl 2.5 mM each dNTP, 0.066 μl UP2 primer at 100 μM, 0.88 μl 50 mM MgSO_4_, 0.44 μl Platinum *Taq* DNA polymerase, and 13.654 μl H_2_O per 1 × volume) at 95 °C for 3 min, 50 °C for 2 min, and 72 °C for 10 min. PCR amplification (95 °C for 3 min and then 20 cycles of 95 °C for 30 s, 67 °C for 1 min, and 72 °C for 6 min 6 s) was performed with master mix (4.1 μl 10 × high-fidelity PCR buffer, 1.64 μl 50 mM MgSO_4_, 4.1 μl 2.5 mM).

The four reactions of each sample were pooled and purified using the Qiagen PCR purification kit (cat. no. 28106) and eluted in 50 μl EB buffer. The samples were selected by testing for genes, Vimentin, N-Cadherin, E-Cadherin, MMP2, and MMP9.

Primers and probes (Supplementary Table 6) were designed with AlleleID (Premier Biosoft) and synthesized by Stem Cell Technology Research Center (Bonyakhte Co). Nucleotide sequences used for design of probe-primers were retrieved from NCBI database and the designed probe-primers were aligned by BLAST to confirm gene specificity.

Each sample was again divided into 4 and a second round of PCR amplification (nine cycles of 98 °C for 3 min, 67 °C for 1 min, and 72 °C for 6 min 6 s) was performed with master mix (9 μl 10 × high-fidelity PCR buffer, 3.6 μl 50 mM MgSO_4_, 13.5 μl 2.5 mM each dNTP, 0.9 μl AUP1 primer at 100 μM, 0.9 μl AUP2 primer at 100 μM, 1.8 μl Platinum *Taq* DNA polymerase, and 59.1 μl H_2_O per 1 × volume). Samples were pooled and purified using Agencourt AMPure XP beads and eluted in 40 μl 1 × low-TE buffer. The universal Proponents also claim that micrometastases are found in all of doubtful patients (G3 cases who had negative lymph nodes by H&E or Pap staining but Positive by Metas-Chip) upon re-examination, and that current histological detection methods may, therefore, be inadequate for identifying metastatic tumor cells in lymph nodes in breast. (PRIMER SEQUENCE).

### Data availability

The authors declare that all the other data supporting the findings of this study are available within the article and its supplementary information files and from the corresponding author upon reasonable request.

## Electronic supplementary material

Supplementary Information

Description of Additional Supplementary Files

Peer Review File

Supplementary Movie 1

Supplementary Movie 2

Supplementary Movie 3

Supplementary Movie 4

Supplementary Movie 5

Supplementary Movie 6

Supplementary Movie 7

Supplementary Movie 8

Supplementary Movie 9
