# Supplementary material for: Metas-Chip precisely identifies presence of micrometastasis in live biopsy samples by label free approach
Source: Nat Commun. 2017 Dec 19;8:2175. doi: 10.1038/s41467-017-02184-x (PMC5736647; doi:10.1038/s41467-017-02184-x)
Supplement: Supplementary file 1 — Supplementary Information [file 41467_2017_2184_MOESM1_ESM.pdf]

Supplementary Information

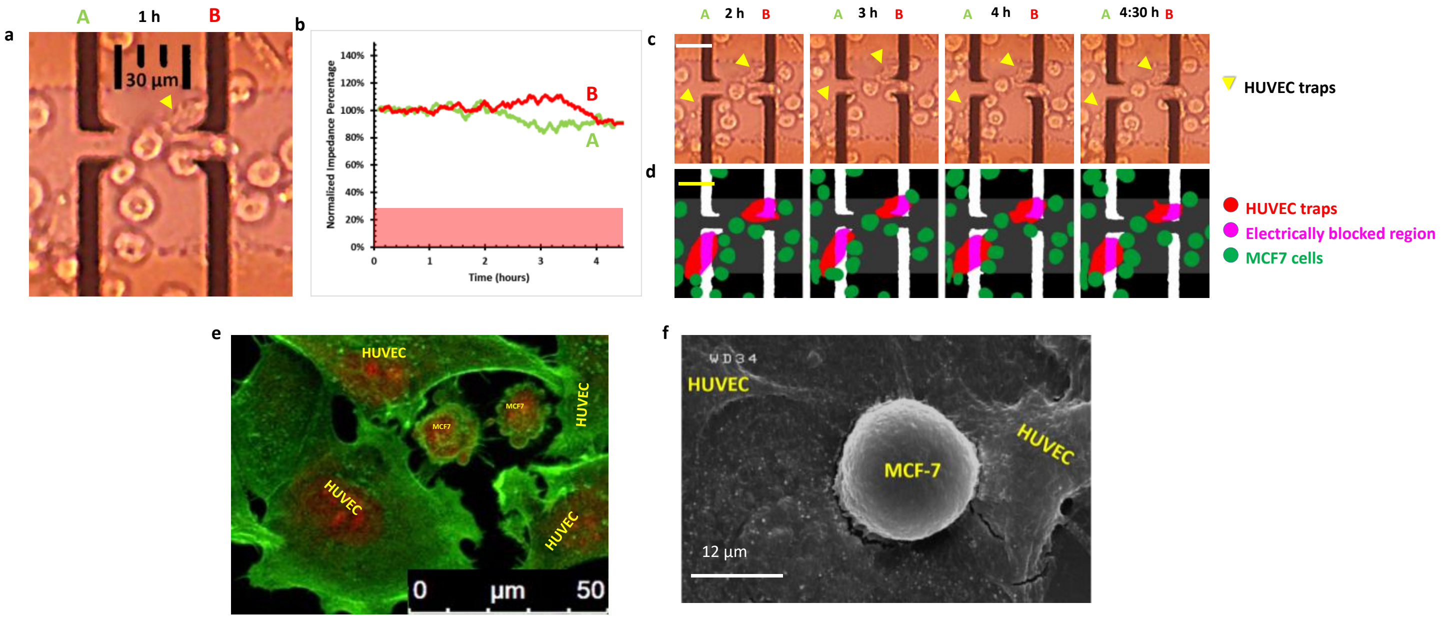

Supplementary Figure 1 - No invasive interaction was observed and recorded for MCF-7 cells in similar time. Cells just attach beneath the HUVEC traps (a) but neither retract them nor induce reductive electrical spikes (b & c). HUVECs maintained their primary location with minor fluctuation during 4.5hr after addition of MCF-7 cells (d). Confocal (e) and FE-SEM image (f) images from interaction between MCF-7 cells and HUVEC trap. Scale Bars in c and d are 30 µm in length.

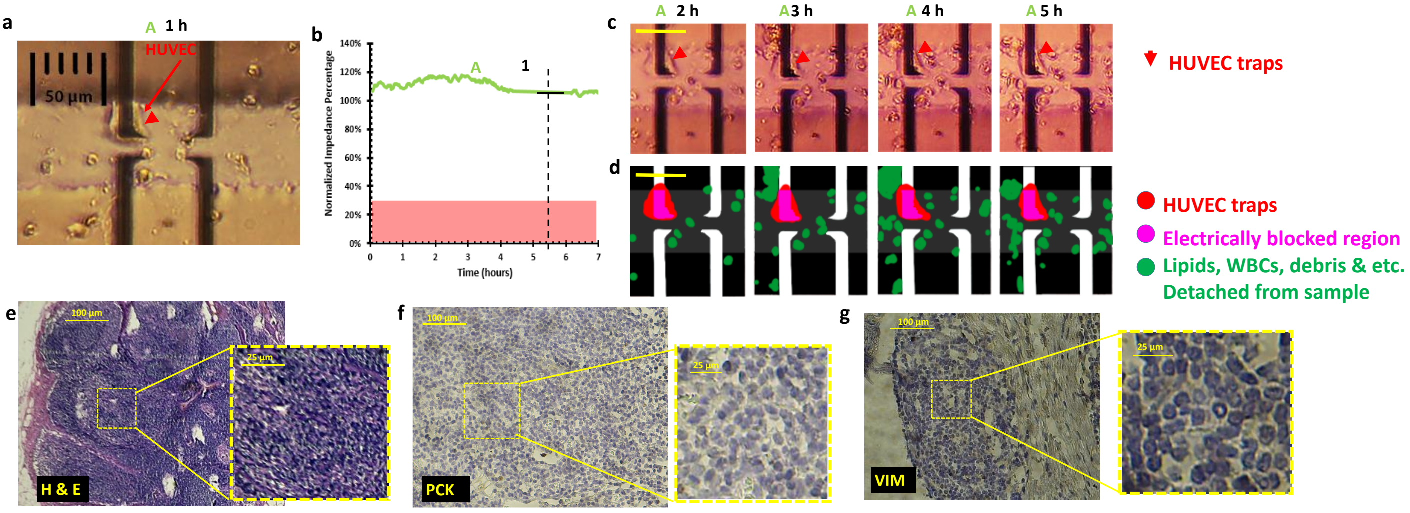

Supplementary Figure 2 - Non metastatic sample images taken from whole slide images of breast tissue. (a) Metas-Chip didn't trap any tumor cell with invasive interaction and no signal reduction was recorded. Some lipid cells, WBCs and debris detached from the lymph biopsy sample could be observed on the sensor without applying any invasive interaction with sensing traps. (c) H&E stained SLNs specimen presented no trace of any metastatic cells. IHC of the patient's SLNs stained by (d) P-CK and (e) vimentin show no expression of malignant markers. Scale Bars in c and d are 50 µm in length.

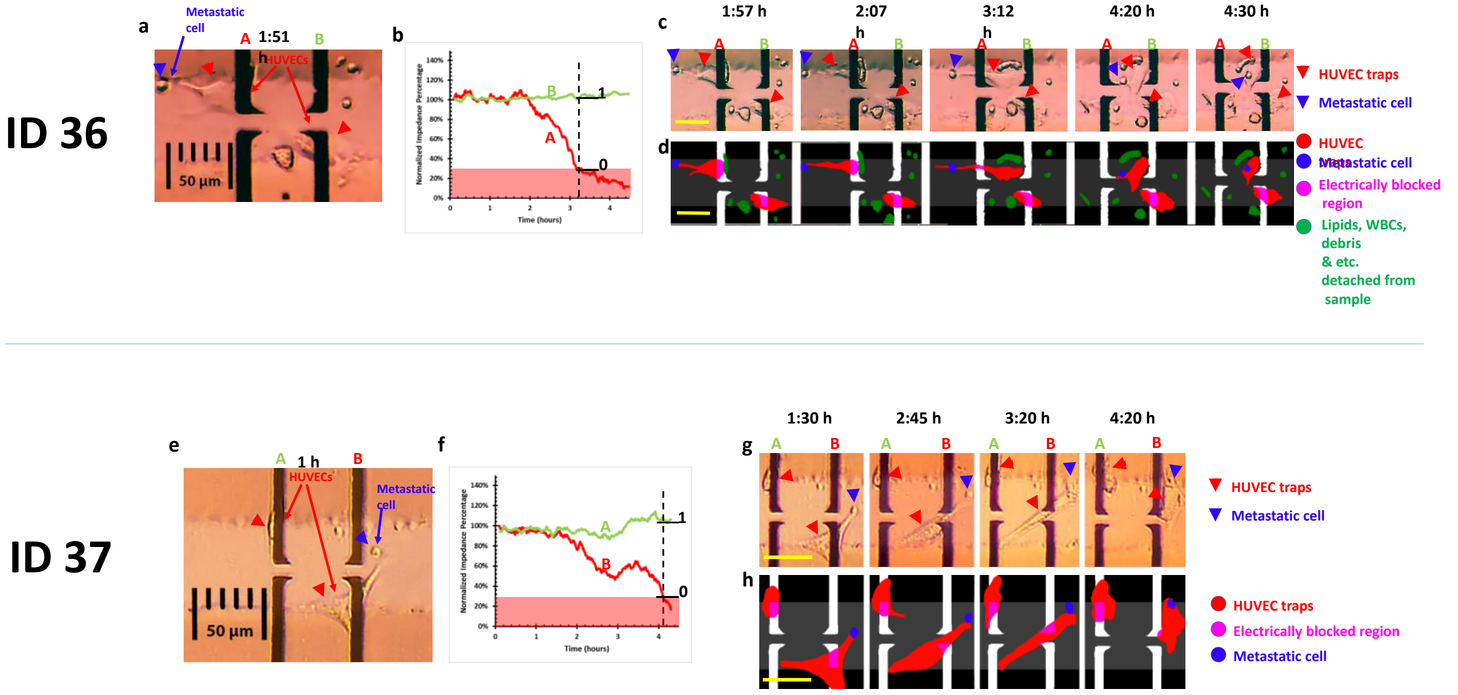

Supplementary Figure 3 – Metas-Chip, histopathological & IHC results of the patients who are suspicious for metastasis. Metas-Chip detected at least one invasive cell (a & e) induced reductive electrical spike in the response of the sensing trap in both of the patients (b & f) which corroborated by time-lapse optical imaging captured by Metas-Chip (c & g). Moreover simultaneous schematics indicate the impedance reduction of the electrodes is correlated by the retraction of the HUVECs (being invaded) from the sensing region (d & h).

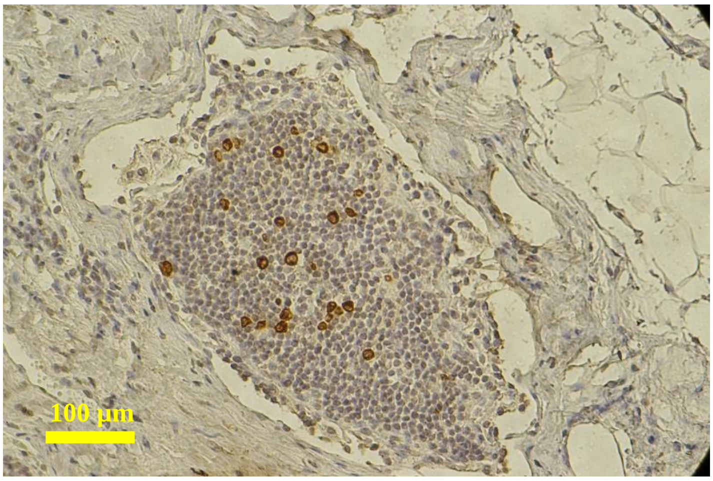

Supplementary Figure 4 - expression of P-CK in the SLN of patient ID:38, whom had been diagnosed as negative by H&E but positive by Metas-Chip

a

b

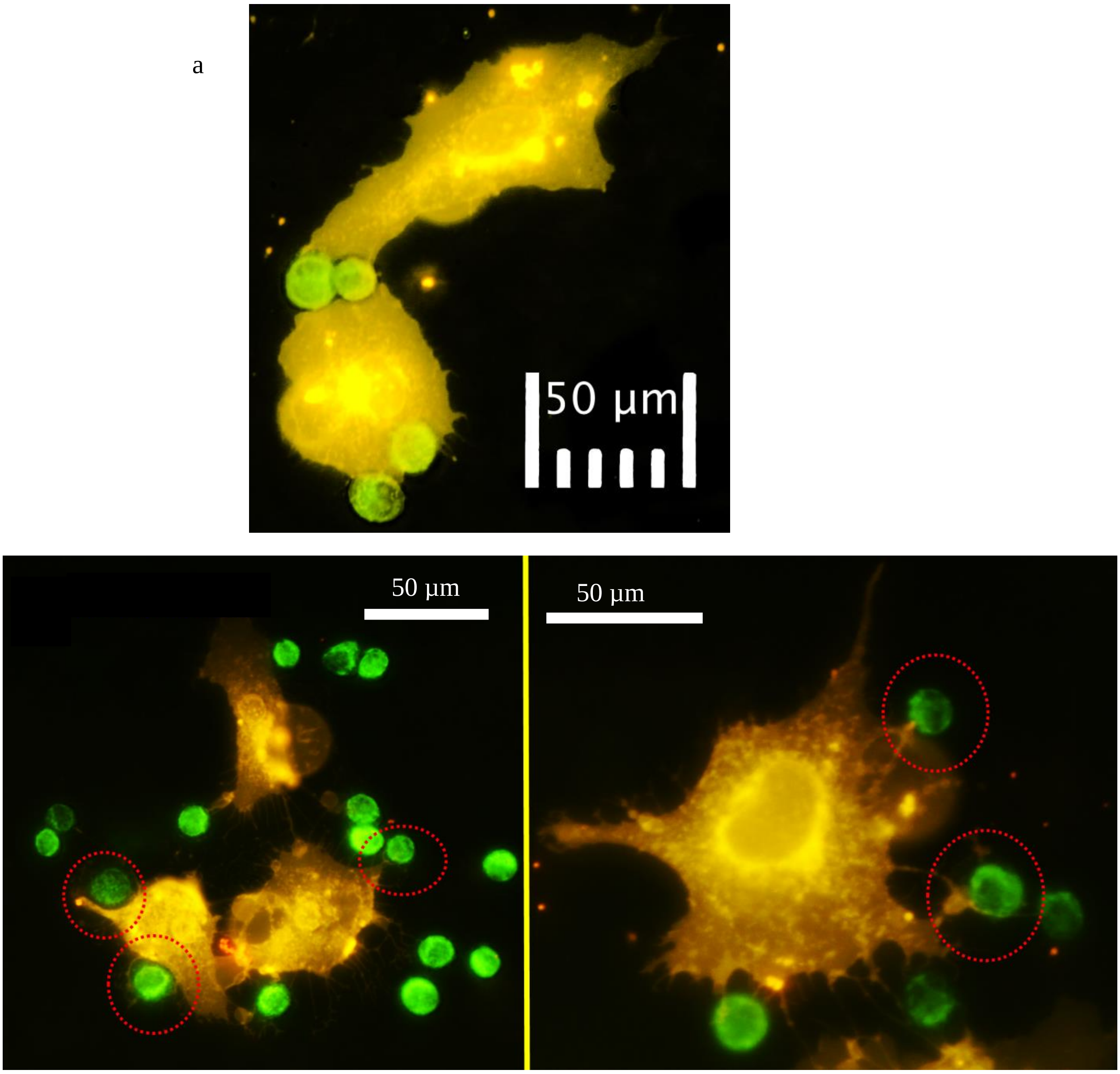

٦٨

Supplementary Figure 5 – a) Epiflourescent image taken from a retracted endothelial cells invaded by two metastatic breast cells revealed the expression of MMP metastatic markers on both of them. b) Entrance of MMP2 expressed metastatic cells to single HUVEC traps. The red dotted circles presents the startment of metastasis.

Non-matching between prognostic marker expression and metastatic ability of cancer cells

Other prognostic markers such as Ki67 aren't indications in metastatic diagnosis . Multivariate analysis showed that Ki-67 expression in the metastases was a superior independent prognostic factor of clinical outcomes compared to that in the primary tumors [1]. In a patient with metastatic breast cancer (detected by Metas-Chip) with lymph nodes involvement, co-staining of biopsy samples for ki67 showed no notable difference from their expression in non-metastatic breast cancer patients cells (Supplementary Supplementary Figure 6).

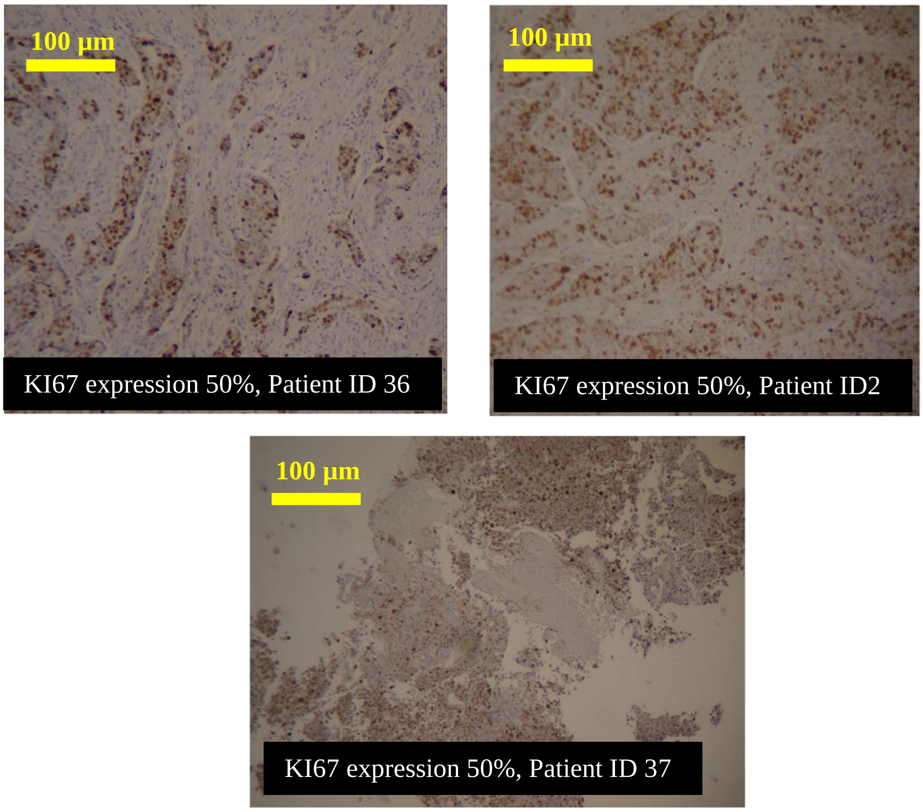

Supplementary Figure 6 - Expression of Ki67 (as prognostic factor) in the breast tumor of the patient, couldn't be an indication for metastasis. Similar expression in two patients with different involvement of cancer cells to their SLNs (ID 36 Vs ID 2).

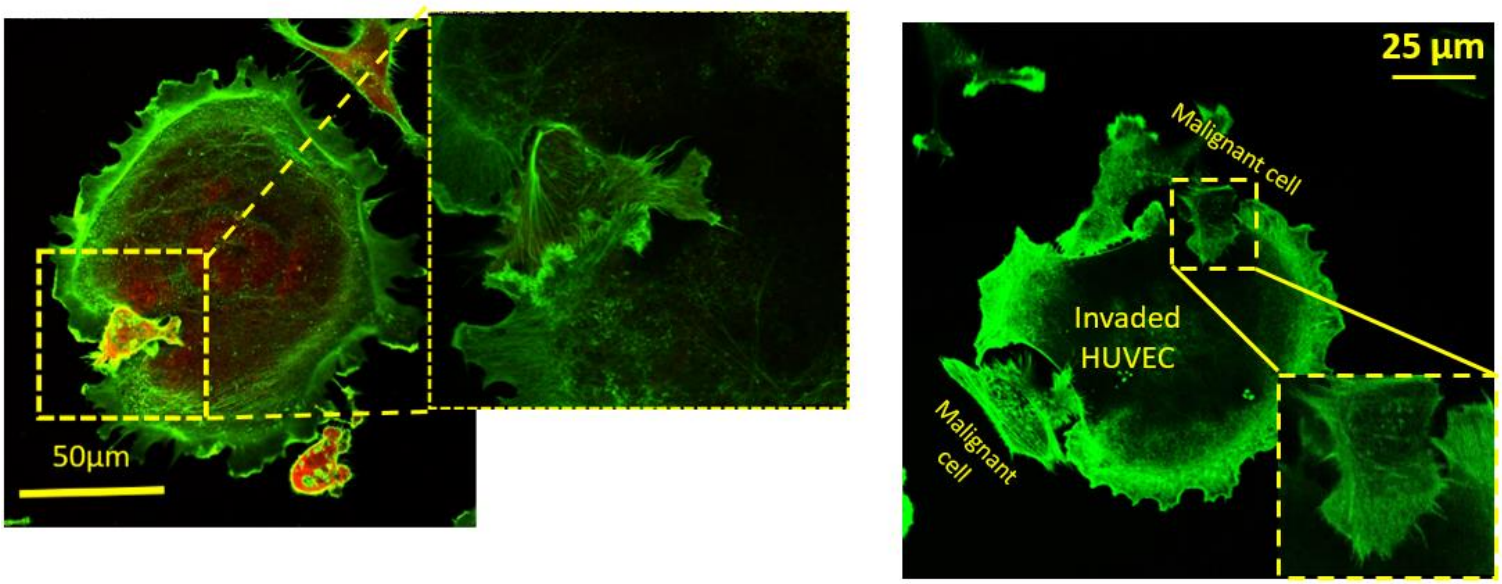

Supplementary Figure 7 - Confocal images of two pairs of metastatic cells during their invasion to two HUVEC traps.

Patient ID	Metas-Chip	Pap stain malignancy	IHC		
			EMA	CK7	PCK
41	✓	✓	✓ (light blue)	✓	✓
42	✓	✓	✓	✓ (light blue)	✓
43	✓	✓	✓	✓	✓
44	✓	✓	✗	✓	✓
45	✓	✓	✓	✓ (light blue)	✓
46	✓	✓	✓	✗	✓
47	✓	✓	✓	✓	✓
48	✓	✓	✓ (light blue)	✗	✓
49	✓	✓	✓	✗	✓
50	✓	✓	✗	✓ (light blue)	✓
51	✓	✓	✓	✓	✓
52	✓	✓	✓	✗	✓
53	✗	✗	✗	✗	✗
54	✗	✗	✗	✗	✗
55	✗	✗	✗	✗	✗
56	✗	✗	✗	✗	✗
57	✗	✗	✗	✗	✗
58	✗	✗	✗	✗	✗
59	✗	✗	✗	✗	✗
60	✗	✗	✗	✗	✗
61	✗	✗	✗	✗	✗
62	✗	✗	✗	✗	✗
63	✗	✗	✗	✗	✗
64	✗	✗	✗	✗	✗
65	✗	✗	✗	✗	✗
66	✗	✗	✗	✗	✗
67	✓	✗	✓ (light blue)	✓ (light blue)	✗
68	✓	✗	✓ (light blue)	✗	✓ (light blue)
69	✓	✗	✓ (light blue)	✗	✗
70	✓	✗	✗	✗	✓ (light blue)

G1 G2 G3

Metas-Chip and Pap legends

✓ Positive

✗ Negative

IHC legend

✓ Many metastatic cells

✓ (light blue) Few metastatic cells

✗ No metastatic cell

Supplementary Table 1 - Metas-Chip, Pap staining and IHC diagnostic results of lymph nodes removed from breast cancer patients by FNA. Expression of Pancytocheratin(PCK), Epithelial Membrane Antigen(EMA) and Cytocheratin-7(CK7) markers were assayed by IHC as a reference diagnosis in FNA samples.

`

Patient ID	Metas-Chip	H&E Invasion	IHC	RT-PCR	
			PCK	E-Cadherin	N-Cadherin
1	✓	✓	✓	2.78	21.56
2	✓	✓	✓	3.45	24.56
3	✓	✓	✓	3.85	36.68
13	✓	✓	✓	4.71	20.72
14	✓	✓	✓	3.15	22.76
15	✓	✓	✓	2.22	28.29
25	✓	✓	✓	2.09	33.81
27	✓	✓	✓	3.68	29.58
28	✓	✓	✓	3.15	23.67
32	✓	✓	✓	2.77	24.03
33	✓	✓	✓	3.75	26.93
36	✓	✓	✓	3.73	29.75
37	✓	✓	✓	3.05	20.38
38	✓	✓	✓	3.37	37.73
39	✓	✓	✓	2.41	23.34
40	✓	✓	✓	4.06	37.17

G1 G2 G3

Metas-Chip and H&E legends

✓ Positive

✗ Negative

IHC legend

✓ Many invasive cancer cells

✓ Few invasive cancer cells

✗ No invasive cancer cell

RT-PCR legend

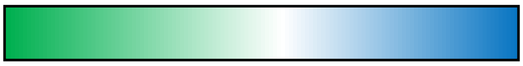

0 1 >10

Supplementary Table 2 - Metas-Chip, H&E, IHC and RT-PCR diagnostic results of breast tumors removed from breast cancer patients by CNB. Expression of Pancytocheratin(PCK) marker was assayed by IHC as a reference diagnosis in the samples. Detection of invasive cells in the CNB of the patients' breast region (diagnosed by both Metas-Chip & pathological assays) has been corroborated by sharper expression of N-Cad transcript.

Patient ID	Metas-Chip	Pap Stain malignancy
41	✓	✓
42	✓	✓
43	✓	✓
57	✗	✗
58	✗	✗
67	✓	✓

G1 G2 G3

Metas-Chip and Pap legend

✓ Positive

✗ Negative

Supplementary Table 3 - Metas-Chip and H&E diagnostic results of breast tumors removed from breast cancer patients by FNA.

Step	Reagent	Time
**1**	95% Alcohol (Fixation)	15-30 minutes
**2**	80% Alcohol	2 minutes
**3**	60% Alcohol	2 minutes
**4**	Distilled Water	5 dips
**5**	Distilled Water	5 dips
**6**	Hematoxylin stain	3 minutes
**7**	Distilled Water	3 minutes
**8**	60% Alcohol	2 minutes
**9**	80% Alcohol	2 minutes
**10**	95% Alcohol	2 minutes
**11**	Orange G Stain	3 minutes
**12**	95% ALcohol	2 minutes
**13**	95% Alcohol	2 minutes
**14**	Eosin Azure Stain	3 minutes
**15**	95% Alcohol	2 minutes
**16**	95% Alcohol	2 minutes
**17**	95% Alcohol	2 minutes
**18**	95% Alcohol	2 minutes
**19**	Absolute Alcohol	2 minutes
**20**	Absolute Alcohol	2 minutes
**21**	Absolute Alcohol	2 minutes
**22**	Absolute Alcohol+Xylene (1:1)	2 minutes
**23**	Xylene	2 minutes
**24**	Xylene	2 minutes
**25**	Xylene	Till clear
**26**	Mount in D.P.X	

Supplementary Table 4 - Procedure of Progressive Papanicolaou Staining Method used for staining of FNA samples

Biospecimen type	- Solid tissue, Core needle biopsy from breast tumor and lymph node<br>- Fine Needle Aspiration
Anatomical site	Breast
Disease status of patients	Breast Cancer
Clinical characteristics of patients	Breast cancer patients with suspicious lymph node to metastasis
Vital State of patients	Alive
Clinical diagnosis of patients	Breast cancer
Pathology diagnosis	Ductal Invasive Carcinoma with different grades with involved or safe sentinel lymph nodes.<br>Threated breast cancer with involved or safe auxiliary lymph nodes
Collection mechanism	Core needle biopsy or Fine needle aspiration
Type of stabilization	For Metas-Chip assaying: DMEM+FBS, 37 °C<br>For Pathological assaying: Fixing in formalin
Type of long-term preservation	Freezing for pathological assay, exposing to RNase & storing in liquid N2 for Real Time PCR
Constitution of preservative	10% neutral-buffered formalin
Storage temperature	37 °C for metas-chip assays, -196 °C for Real Time PCR
Storage duration	5 hours for metas-chip assays, 8 months for Real Time PCR
Shipping temperature	37 °C for metas-chip assays, -196 °C for Real Time PCR
Composition assessment & selection	Patients candidate for core needle biopsy with suspicious lymph nodes

Supplementary Table 5 - Quick-Reference BRISQ Summary/Checklist

Name	Primer-probe	Sequence (5'-3')	Tm (ºC)	Tm set up	Amplicon size (bp)
**N-Cadherin**	R	TGCCTTCATCACCAAACATTTGGC	58.5	60	115
**N-Cadherin**	F	GCTGGCCGTAAACTGCTTTGTG	58.6	60	115
**E-Cadherin**	R	GTACTCGCTCATAGGATGGTAGG	56.4	60	136
**E-Cadherin**	F	GGTGCTGGTCTGTGTTCTGG	58.3	60	136
**Vimentin**	R	GGGTTCCGCTGGATCAAGACC	58.2	60	138
**Vimentin**	F	CCTGGCCGTGCTAGACAATGG	58.5	60	138
**Beta2M**	F	ATG CCT GCC GTG TGA AC	55	60	91
**Beta2M**	R	ATC TTC AAA CCT CCA TGA TG	54	60	91
**MMP2**	F	GCT CGT GCC TTC CAA GTC	58.4	59	136
**MMP2**	R	AGT CCG TCC TTA CCG TCA A	57.3	63	181
**MMP9**	F	CGG ACC AAG GAT ACA GTT TGT	59.4	60	116
**MMP9**	R	CTC AGT GAA GCG GTA CAT AGG	61.3	63	181

Supplementary Table 6 - Oligonucleotide primer and probe sequences used in the present study.
