# Supplementary material for: Metas-Chip precisely identifies presence of micrometastasis in live biopsy samples by label free approach
Source: Nat Commun. 2017 Dec 19;8:2175. doi: 10.1038/s41467-017-02184-x (PMC5736647; doi:10.1038/s41467-017-02184-x)
Supplement: Supplementary file 2 — Description of Additional Supplementary Files [file 41467_2017_2184_MOESM2_ESM.pdf]

Description of Additional Supplementary Files

File Name: Supplementary Movie 1
Description: Invasion of a metastatic cluster to a vascular cell

File Name: Supplementary Movie 2
Description: Selective attachment of single HUVECs on sensing traps by DEP

File Name: Supplementary Movie 3
Description: Preferred attachment of a metastatic cell on the vascular cell

File Name: Supplementary Movie 4
Description: Operation of MetasChip on a biopsy sample

File Name: Supplementary Movie 5
Description: Interaction of non-malignant lymph node with HUVEC

File Name: Supplementary Movie 6
Description: Interaction of WBCs of a healthy donator with HUVEC

File Name: Supplementary Movie 7
Description: Interaction of malignant lymph node sample, resected by FNA, with HUVEC

File Name: Supplementary Movie 8
Description: Identification of a metastatic cell by a WBC without suppressing its invasion to the HUVEC

File Name: Supplementary Movie 9
Description: Migration of a WBC among HUVECs
