# Supplementary material for: Metas-Chip precisely identifies presence of micrometastasis in live biopsy samples by label free approach
Source: Nat Commun. 2017 Dec 19;8:2175. doi: 10.1038/s41467-017-02184-x (PMC5736647; doi:10.1038/s41467-017-02184-x)
Supplement: Supplementary file 3 — Peer Review File [file 41467_2017_2184_MOESM3_ESM.pdf]

Reviewer #2 (Remarks to the Author):

This is a very interesting and innovative study reporting a new method to detect micrometastasis in tissue samples. Although the method has promise, as presented, the study has limitations.

The importance of the process of EMT in metastasis is oversimplified and overstated. It has not been shown that EMT is a required step for metastasis in all cancers. The authors state that there are no false positive results, however, they have not investigated this possibilities extensively, as should be performed to establish a new method. For example, macrophages and lymphocytes may intravasate and extravasate, and may mimic cancer cells in this system representing a potential source of false positive results. The study involves a very small number of samples, and no separate validation cohorts. Whether the system can quantify the number of cells and/or the size of the metastatic foci would be important clinically.

Reviewer #3 (Remarks to the Author):

I am extremely concerned that the manuscript has a very commercial feel to it and appears to over-simplify singular features that make a cell metastatic and an electrical characteristic that can be measured. My own experience with S100A8 research in the past 10 years has proven challenging to single out S100A8 as a predictive or therapeutic target (moreover, a number of the prognostic studies are underpowered.

Whereas the methodology appears to be reasonable, 20 patients is far too small a sample size in order to merit publication.

Secondary comments:
1. It would be helpful to know if the cells they captured would in fact produce metastasis in mice or if the cells are no longer viable after testing.

2. If one could indeed show this in mice, one might have additional markers to look at (as the authors mentioned.)

Decision: Reject

Reviewer #4 (Remarks to the Author):

The manuscript entitled "Metas-Chip identifies metastasis in biopsy samples of cancer patients faster 1 and more precise than conventional histopathological and immunohistochemical methods" is trying to demonstrate a microelectronic biochip (named Metas-Chip) that allows to detect the presence of metastasis in unprocessed samples. It was reported that the metastatic cells in the biopsied samples retract the traps's HUVEC, which induces sharp changes in electrical response. The metastasis in assayed breast cancer patients was identified with the accuracy of 100% using the Metas-Chip in less than 5 hours. In my opinion, the scientific merit and originality are enough to be published in this journal if the following concerns are addressed.

Major concerns
1. More detailed information regarding biopsy preparation is required. For example, what size the biopsy should be? where the biopsy should put in the biochip?
2. In Supplementary Figure 1, the effect of MCF7 on HUVEC was presented. However, more negative control experiments should be demonstrated besides MCF 7. In addition, please explain the number of experiments as showed in Supplementary Figure 1b
3. The manuscript claimed that "other types of the cells existed in the biopsied tissue like non-invasive epithelial cells, peripheral lipids and blood cells don't apply invasive interaction by HUVEC

traps so wouldn't be captured by the Metas-Chip". Although, the effect of lipid, WBCs and debris on HUVEC was showed in Supplementary figure 2, please explain the number of experiments as showed, as well as whether macrophages or immune cells caused the retraction of HUVEC.

4. In figure 4, samples from 20 patients were analyzed. Their clinical information was needed. For example, what type of breast cancers are they? What stage? Whether are there correlation between the detection sensitivity and stage?

5. Besides breast cancers, the Metas-Chip could be applicable to other types of cancers, such as lung cancers, etc.

Minor concerns

1. It is difficult to read the characters in figure.
2. Rewrite the legend in Figure 1.

Reply to referees

Thanks to referees' careful consideration, many investigations and discussions were added to the paper which significantly improved the manuscript. I hope the replies and revised manuscript would be convincing.

Best Regards

Reviewer #2 (Remarks to the Author):

"This is a very interesting and innovative study reporting a new method to detect micrometastasis in tissue samples. Although the method has promise, as presented, the study has limitations.

***Q**: The importance of the process of EMT in metastasis is oversimplified and overstated. It has not been shown that EMT is a required step for metastasis in all cancers. "*

Reply:

Thank you for your constructive comment. If the concern is about the first paragraph of our introduction, we only referred to other reports [1] stated on the prerequisite of EMT in metastatic cells. However we modified the introduction due to your query.

But in the case of the patients diagnosed as malignant lymph nodes by MetasChip, we found the trace of the cells expressed Vimentin (as mesenchymal cancer marker) in IHC images taken from their samples.

Of course we won't extend EMT as a prerequisite for metastasis in all types of cancer.

Moreover we cited to some other references that the metastasized cancer cells might be found in hybrid epithelial-mesenchymal phenotypes [2]. On the other hand the lymph of the patients from G3 (diagnosed as invasive by MetasChip but not diagnosed in H&E) expressed meaningful level of metastatic associated markers such as N-Cadherin and Vimentin in RT-PCR assay.

Q: *The authors state that there are no false positive results, however, they have not investigated this possibilities extensively, as should be performed to establish a new method. For example, macrophages and lymphocytes may intravasate and extravasate, and may mimic cancer cells in this system representing a potential source of false positive results.*

Reply:

This is a valuable query. We already stated that no invasive detachment or membrane blebbing (as an indication for cell death [3] of HUVEC sensing traps was observed in their interaction with non-cancerous lymph nodes or benign lymphadenopathies (which themselves contain a plenty of lymphocytes and macrophages because of inflammation induced by primary breast cancer) [4].

However due to your query and for more clarification we added a detailed comprehensive discussion based on both recently published nature review papers ([5]: *Nature Reviews Immunology ,2015; doi:10.1038/nri3908]: "How leukocytes cross the vascular endothelium"*)([6]: *Nature Reviews Cancer vo13 ,2013 , pp 858: "Crossing the endothelial barrier during metastasis"*). Moreover we extended our experiments on non-malignant lymph nodes and immune blood cells in interaction with HUVEC traps through cytopathological, confocal (FigureR 6) and time lapse imaging (FigureR 3a, b). Which is added to the paper. Our findings exclude any immune cell (macrophages and lymphocytes)-HUVEC interaction that could trigger a false positive HUVEC trap response. Here we discuss our findings:

Leukocytes in turn instruct endothelial cells to open a path for transmigration. [5]

Danger signals such as inflammation stimulate resident cells of the innate immune system, such as mast cells, macrophages and dendritic cells, which leads to the secretion of cytokines and other pro-inflammatory mediators that activate nearby endothelial cells of the microvasculature. Thereby, a cascade of events is triggered that enables leukocytes to recognize the vascular endothelium and to interact with them through a series of steps known as capturing, rolling, leukocyte arrest, crawling to sites of exit and transmigration through the barriers of endothelial cells, pericytes and the basement membrane [5](FigureR 1).

The important point is that with the assistance of LBRC and Actomyosin, the endothelial cells reseal themselves after extravasation of immune blood cells [5].

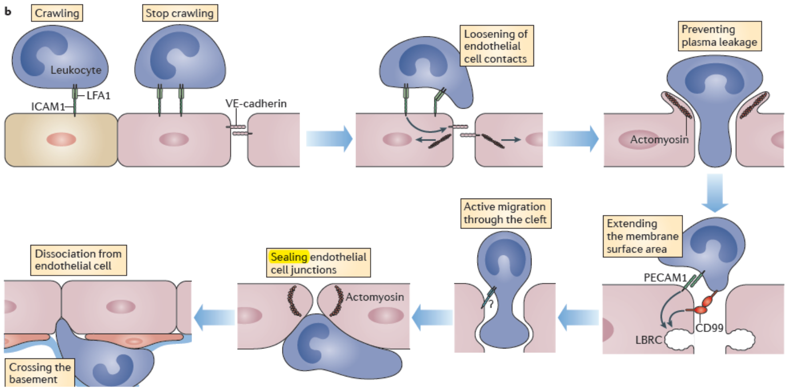

FigureR 1- The diapedesis process requires many functions mediated by leukocytes and endothelial cells: stopping intraluminal crawling at suitable exit sites; loosening of endothelial cell contacts; preventing plasma leakage; extending the membrane surface area at endothelial cell junctions through mobilization of the lateral border recycling compartment (LBRC); active leukocyte migration through the junctional cleft; and sealing of the junction after diapedesis. Finally, leukocytes dissociate from endothelial cells followed by transmigration through the

basement membrane. CD99L2, CD99 antigen-like protein 2; ESAM, endothelial cell-selective adhesion molecule; ICAM, intercellular adhesion molecule; JAM, junctional adhesion molecule; LFA1, lymphocyte function-associated antigen 1; PECAM1, platelet endothelial cell adhesion molecule 1; VCAM1, vascular cell adhesion molecule 1. Secretion of such molecules indicated the pathway matching between HUVECs and vacation of blood cell[5]

Each of the markers and macromolecules have a known role in mediating the intra/extravasation of blood cells without perturbing or induction of membrane blebbing in the HUVECs. As an example:

CD99 was identified as a diapedesis-mediating receptor through the study of human CD99-specific antibodies that blocked monocyte migration through endothelial cell monolayers9. Similarly to PECAM1, CD99 was found on both monocytes and endothelial cells and was required on both cell types for diapedesis. Antibodies specific for mouse CD99 also inhibited lymphocyte entry into inflamed skin and neutrophil recruitment into the inflamed peritoneum [5]

The fact that the endothelial barrier can stay sealed despite the transmigration of leukocytes is remarkable, and it is likely that the endothelial actomyosin system is required to keep junctions tight while they enclose the diapedesing leukocyte. So no pathological phenotypes or apoptotic/necrotic pathways would induce in endothelial cells during intra/extravasation of immune blood cells (FigureR 2) [5].

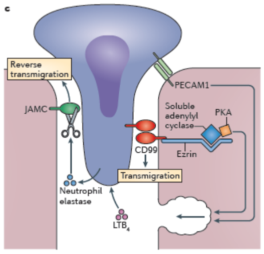

FigureR 2 - Diapedesing neutrophils trigger the mobilization of lateral border recycling compartment (LBRC) vesicles to the junctional plasma membrane of endothelial cells, which increases the membrane surface area at such sites. This effect is initiated by platelet endothelial cell adhesion molecule 1 (PECAM1) and CD99, with the latter signalling through the ezrin-located soluble adenylyl cyclase and protein kinase A (PKA). The leukocyte transmigration process is made irreversible by junctional adhesion molecule C (JAMC) Diapedesing neutrophils trigger the mobilization of lateral border recycling compartment (LBRC) vesicles to the junctional plasma membrane of endothelial cells, which increases the membrane surface area at such sites. This effect is initiated by platelet endothelial cell adhesion molecule 1 (PECAM1) and CD99, with the latter signalling through the ezrin-located soluble adenylyl cyclase and protein kinase A (PKA). The leukocyte transmigration process is made irreversible by junctional adhesion molecule C (JAMC) [5]

Movement of leukocytes through the endothelial cells may be supported by a multivascular compartment, the lateral border recycling compartment (LBRC), inside endothelial cells. It was reported that this compartment is directly connected to the plasma membrane and readily mobilized to the cell surface during leukocyte diapedesis, thereby helping to accommodate the body of the transmigrating leukocytes without entrance of endothelial cells to pathological phenotype [5].

Researcher findings reveal that the cancer cells secrete many proteins to perturb the HUVECs during extravasation [2, 6]. Also cancer cells trace many markers, secreted by HUVECs, to identify and invade the endothelial barriers [6].

Over the past few years, intravital imaging studies have revealed some of the mechanisms that underlie intravasation in vivo; for example, breast cancer cells in xenograft tumors have been shown to move directionally towards blood vessels[6].

Cancer cell extravasation usually occurs in small capillaries, where the cells can be physically trapped by size restriction and can then form stable attachments to endothelial. Meanwhile the leukocytes and macrophages just slide and transmit through endothelial cells by either paracellular or transcellular migrations [6].

A dynamic regulation of the endothelium by cancer cells through the formation of some membrane bridges was observed. (FigureR 6a)

The communication between the tumor cell and the endothelium upregulates markers associated with 'metastatic hijack', in which cancer cell-induced transformation of healthy endothelium into pathological endothelium and resulted in membrane blebbing and retraction of HUVECs sensing traps which was observed in time lapse and confocal images (FigureR 3c, Supplementary Video S7). Such evidence wasn't observed in interaction non-malignant lymph nodes or WBCs with the traps (FigureR 3a,b, Supplementary Video S5 & S6).

It is known cancer cells induce pathological phenotypes to all of the cells being invaded (such as vascular cells) during metastasis [2].

It has been reported that metastatic cells were found to preferentially form heterotypic connections to secondary tissues during metastasis [2], in our opinion, this might be the invadopodias we observed and showed by confocal (FigureR 6a)and Pro MMP2 based immunoflorescent images in our report (Figure 5 in main article).

Neuronal cadherin (N-cadherin; also known as cadherin 2) is another receptor that is involved in the attachment and invasion of cancer cells [6]. This receptor has no role in intra/extravasation of imuno blood cells through endothelial barrier [5]. We showed over expression of N-Cadherin in malignant lymph nodes diagnosed by MetasChip meanwhile the expressing levels were low in safe lymph nodes (Table 1).

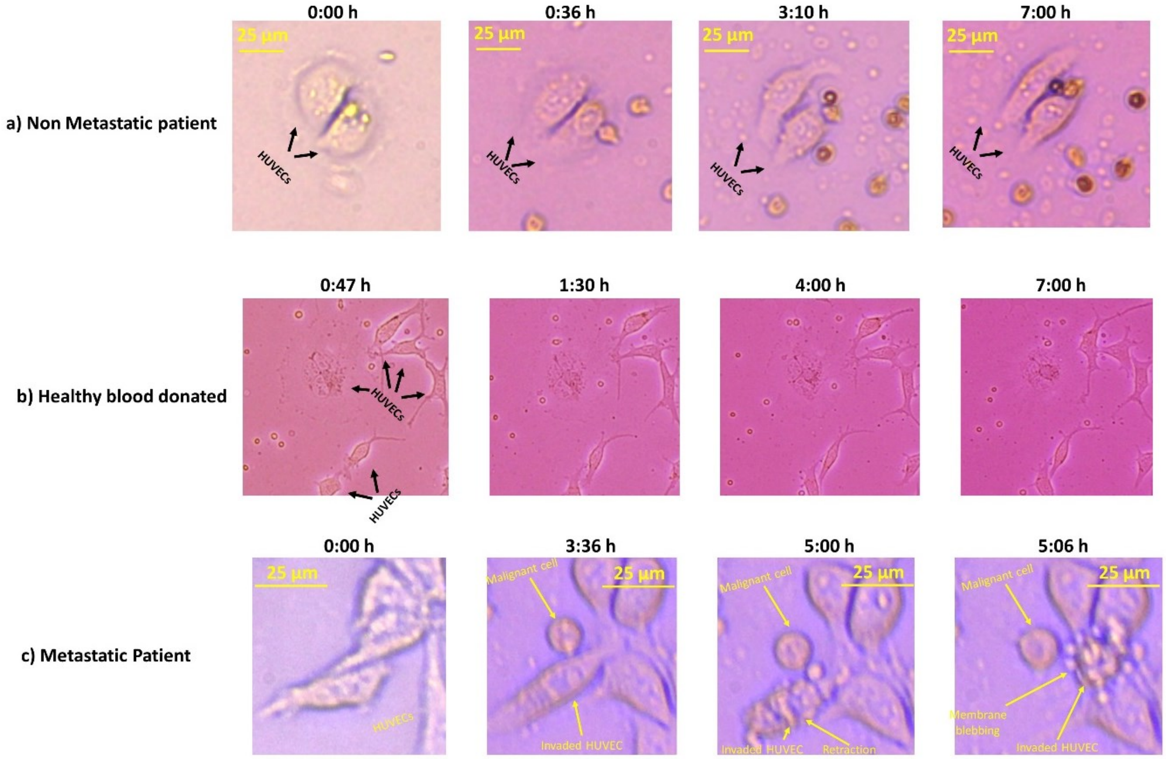

FigureR 3 – Time lapse images of a)Non-metastatic lymph b) Healthy blood and c) metastatic lymph samples in interaction with single HUVECs. HUVEC retraction and membrane blebbing was only induced by the metastatic cell (c). Related Supplementary videos: (g) S6 (h) S7 (i) S8

In summary, those references revealed that only cancer cells induce pathological perturbation into vascular barrier during invasion meanwhile the vascular cells can reseal themselves after intra/extravasation of non-cancerous immune blood cells. The reports indicated that transmigration of

immune cells through vascular barrier is a preprogrammed process with known signaling pathways for endothelial cells [5] meanwhile invasion of cancer cells is a non-programmed process and a non-desired happening for vascular endothelial cells which induces pathological phenotypes in them. This was also seen *in vitro* by retraction and membrane blebbing of HUVEC traps in MetasChip. We observed such pathological transformation by membrane blebbing and retraction of HUVEC traps after being invaded by cancer cells (cell line and patients' samples) (FigureR 3.c) (Supplementary Video S7 & S8) and those perturbations were absent for HUVECs being interacted by safe lymph (Supplementary Video S5, FigureR 3a) and normal WBCs (Supplementary Video S6, FigureR 3.b).

Giemsa cytopathological images also showed the HUVECs being perturbed by various MDA-MB468 (FigureR 4b-d).

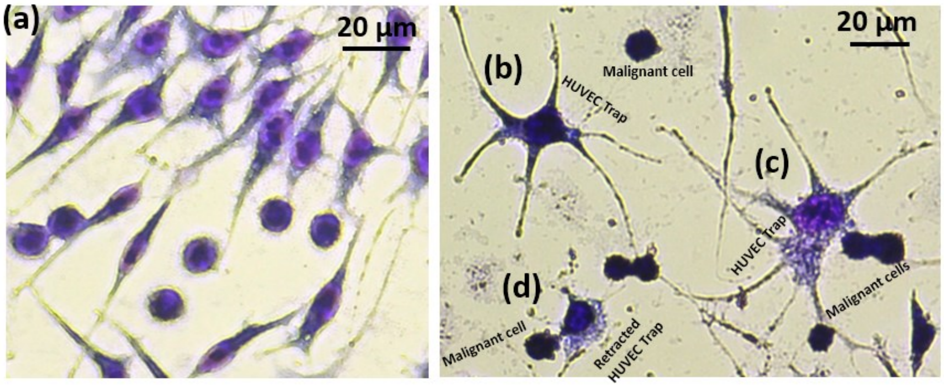

FigureR 4 - a) HUVEC traps after 7hr of interaction by Immuno blood cells b) Non invaded trap c) Start of invasion d) Invasion, start of retraction Perturbation of HUVECs due to invasion of metastatic cells were highlighted by additional confocal images

Similar images were taken from the interaction of non-malignant lymph nodes and HUVECs (FigureR 4a) no retraction was observed in the structure of interacted HUVECs.

Trace of transcellular and paracellular transmigrations of leukocytes during intra/extravasation through endothelial vascular layer without inducing any perturbation were reported (FigureR 5) [7]. We observed similar confocal images from non-retracted endothelial layer after TC and PC of blood immune cells (from non-metastatic patients) (FigureR 6.e & f).

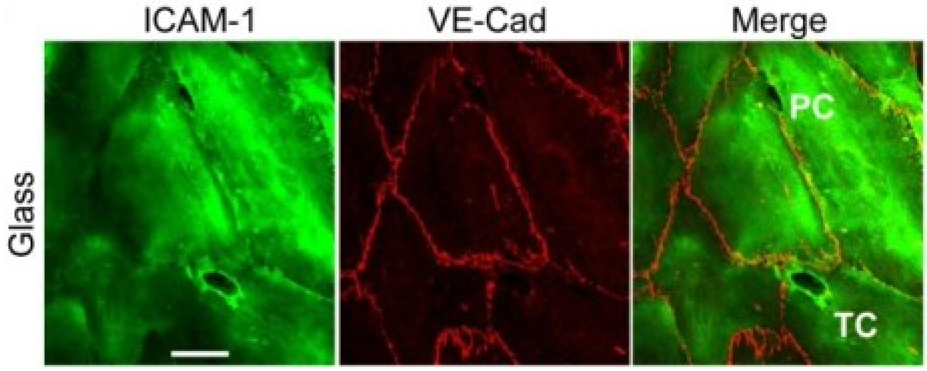

FigureR 5 - Representative confocal fluorescence images of cells taking the paracellular (PC) and transcellular (TC) routes. The migrating NK cells appear as small dark holes surrounded by intense anti-ICAM-1 staining, and the endothelial cell-cell junctions are visualized by anti-VE-cadherin staining. The endothelial cell substrate was glass in the upper panel and soft substrate (polyacrylamide) in the lower panel. Scale bar =10µm.[7]

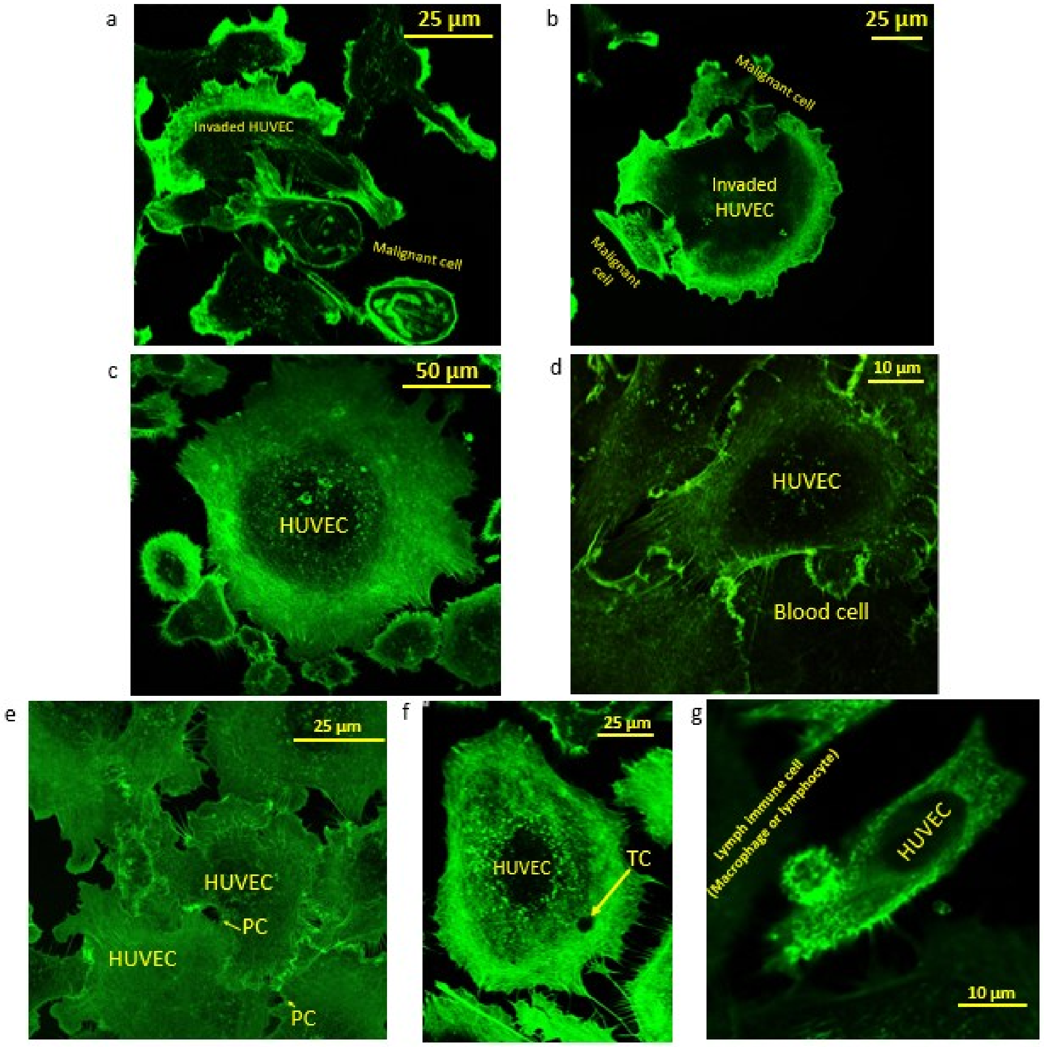

FigureR 6 - (a) Confocal images from interaction of two individual malignant cell samples by HUVEC traps. Retraction of the membrane and gins of membrane blebbing could be observed (b) Perturbation of HUVECs due to invasion of metastatic cells were highlighted by additional confocal images. (c) Similar images were taken from the interaction of non-malignant lymph nodes and HUVECs and no retraction was observed in the structure of interacted HUVECs. (e) The trace of the hole produced by transcellular migration of leukocytes could be observed. (f)Similar confocal images from non-retracted endothelial layer after TC of blood immune cells were reported by others. (g) Moreover attachment of an immuno cell existed in non-malignant lymph was observed but no retraction or membrane blebbing of the HUVECs was happened even after 6 hours.

Hence we observed no false positive result. The additional experiments on leukocytes and non-malignant lymph nodes were added to the paper.

Q: *The study involves a very small number of samples, and no separate validation cohorts.*

Reply:

During the period of submission and revision, we experimented the MetasChip on more than 50 additional breast cancer patients. 20 of the added samples were CNB and the others were Fine Needle Aspiration (FNA) resected from the ALN of breast cancer patients removed lymph nodes. The results presented below indicates higher precision of MetasChip vs. Papanicolaou staining (conventionally used for FNA samples). 4 of the patients were missed by pap staining but diagnosed as involved LNs in MetasChip.

Investigating PCK, CK-7 and EMA based IHC corroborated the accuracy of MetasChip.

Moreover in all of suspicious CNB and FNA samples (positively scored by MetasChip, Negative by H&E or Pap) (9/70), IHC confirmed the accuracy of MetasChip. Again we didn't observe any patient whom positively scored by H&E or Pap assays but scored negative in Metas-chip.

CNB Lymph samples

Patient ID	MetasChip	H&E metastasis	IHC		RT-PCR			
			Vim	PCK	Vimentin	N-Cadherin	MMP2	MMP9
1	✓	✓	✓	✓	41.26	15.64	15.11	17.66
2	✓	✓	✓	✓	12.84	38.05	11.5	15.7
3	✓	✓	✓	✓	18.28	21.11	19.77	8.32
4	✓	✓	✓	✓	15.076	32.85	9.95	15.23
5	✓	✓	✓	✓	42.81	28.23	13.97	8.65
6	✓	✓	✓	✓	40.98	22.43	17.09	9.78
7	✓	✓	✓	✓	13.43	20.62	6.59	9.15
8	✓	✓	✓	✓	56.84	28.92	8.01	10.26
9	✓	✓	✓	✓	28.56	31.58	11.41	13.46
10	✓	✓	✓	✓	42.75	27.55	6.42	18.18
11	✓	✓	✓	✓	25.79	36.4	17.85	7.47
12	✓	✓	✓	✓	11.48	27.41	11.18	17.68
13	✓	✓	✓	✓	11.17	21.32	9.37	9.15
14	✓	✓	✓	✓	40.54	23.23	19.02	13.3
15	✓	✓	✓	✓	55.93	38.24	17.98	10.38
16	✓	✓	✓	✓	20.25	30.99	14.25	10.92
17	✓	✓	✓	✓	23.61	38.19	11.21	5.64
18	✓	✓	✓	✓	30.96	24.49	12.47	13.63
19	✓	✓	✓	✓	33.97	34.72	5.3	15.81
20	✓	✓	✓	✓	11.93	28.26	18.95	13.2
21	✓	✓	✓	✓	34.34	23.9	7	5.82
22	✓	✓	✓	✓	49.8	23.58	12.72	16.13
23	✓	✓	✓	✓	56.74	37.46	14.01	12.8
24	✓	✓	✓	✓	15.14	38.46	14.83	17.71
25	✓	✓	✓	✓	4.67	18.47	16.74	12.87
26	✓	✓	✓	✓	19.41	32.14	11.74	12.45
27	✗	✗	✗	✗	0	2.03	2.6	0.53
28	✗	✗	✗	✗	0.49	1.11	3.76	0.5
29	✗	✗	✗	✗	0.12	0.83	1.77	0.28
30	✗	✗	✗	✗	0.32	0.97	1.56	0.26
31	✗	✗	✗	✗	0.83	2.12	2.11	0.46
32	✗	✗	✗	✗	0.64	2.52	4.28	0.51
33	✗	✗	✗	✗	2.3	4.37	5.6	1.31
34	✗	✗	✗	✗	0.93	1.32	3.79	0.3
35	✗	✗	✗	✗	0.9	1.83	3.04	0.36
36	✓	✗	✓	✓	3.16	2.5	18.25	11.47
37	✓	✗	✓	✓	4.84	14.25	15.28	2.21
38	✓	✗	✓	✓	2.04	1.22	5.32	6.91
39	✓	✗	✓	✓	3.28	14.8	12.62	7.74
40	✓	✗	✓	✓	2.66	1.21	18.92	7.19

G1 G2 G3

MetasChip & H&E & Pap legend
✓ Positive
✗ Negative

IHC legend
✓ Many metastatic cells detected
✓ Few metastatic cells detected
✗ No metastatic cell detected

FNA Lymph samples

Patient ID	MetasChip	Pap Stain malignancy	IHC		
			EMA	CK7	PCK
41	✓	✓	✓	✓	✓
42	✓	✓	✓	✓	✓
43	✓	✓	✓	✓	✓
44	✓	✓	✗	✓	✓
45	✓	✓	✓	✓	✓
46	✓	✓	✓	✗	✓
47	✓	✓	✓	✓	✓
48	✓	✓	✓	✗	✓
49	✓	✓	✓	✗	✓
50	✓	✓	✗	✓	✓
51	✓	✓	✓	✓	✓
52	✓	✓	✓	✗	✓
53	✗	✗	✗	✗	✗
54	✗	✗	✗	✗	✗
55	✗	✗	✗	✗	✗
56	✗	✗	✗	✗	✗
57	✗	✗	✗	✗	✗
58	✗	✗	✗	✗	✗
59	✗	✗	✗	✗	✗
60	✗	✗	✗	✗	✗
61	✗	✗	✗	✗	✗
62	✗	✗	✗	✗	✗
63	✗	✗	✗	✗	✗
64	✗	✗	✗	✗	✗
65	✗	✗	✗	✗	✗
66	✗	✗	✗	✗	✗
67	✓	✗	✓	✓	✗
68	✓	✗	✓	✗	✓
69	✓	✗	✓	✗	✗
70	✓	✗	✗	✗	✓

CNB Tumor Samples

Patient ID	MetasChip	H&E Invasion	IHC	RT-PCR	
			PCK	E-Cadherin	N-Cadherin
1	✓	✓	✓	2.78	21.56
2	✓	✓	✓	3.45	24.56
3	✓	✓	✓	3.85	36.68
13	✓	✓	✓	4.71	20.72
14	✓	✓	✓	3.15	22.76
15	✓	✓	✓	2.22	28.29
25	✓	✓	✓	2.09	33.81
27	✓	✓	✓	3.68	29.58
28	✓	✓	✓	3.15	23.67
32	✓	✓	✓	2.77	24.03
33	✓	✓	✓	3.75	26.93
36	✓	✓	✓	3.73	29.75
37	✓	✓	✓	3.05	20.38
38	✓	✓	✓	3.37	37.73
39	✓	✓	✓	2.41	23.34
40	✓	✓	✓	4.06	37.17

FNA Tumor Samples

Patient ID	MetasChip	Pap Stain malignancy
41	✓	✓
42	✓	✓
43	✓	✓
57	✗	✗
58	✗	✗
67	✓	✓

TableR 1 - Metas-Chip, H&E, Pap, IHC and RT-PCR diagnostic results of breast tumors & Lymph nodes removed from breast cancer patients by CNB & FNA. Expression of Vimentin(Vim) , Pancytocheratin(PCK), Epithelial Membrane Antigen(EMA) and Cytocheratin-7(CK7) Markers were assayed by IHC as a reference diagnosis in CNB & FNA samples. Detection of metastasis in each assay is correlated with expression levels of transcripts associated with the presence of malignancy in the lymph region such as Vim, N-Cad, MMP2, and MMP9. The trace of transcripts in suspicious SLNs are sharply distinguishable than safe samples which indicates the accuracy of Metas-Chip (I). Detection of invasive

cells in the CNB & FNA of the patients' breast region (diagnosed by both Metas-Chip & pathological assays) has been corroborated by sharper expression of N-Cad transcripts (II).

Validation cohorts in our study were standard cell lines (normal, cancerous and malignant breast cell lines) and the known non-malignant and malignant lymph nodes assayed by multilevel IHCs and RT-PCR from the patients.

As the MetasChip could be so promising in improving the reliability of FNA tests, we hope to increase the interests on FNA based sampling for MetasChip to reduce the pain and side effects induced on patients during CNB sampling.

Q: *Whether the system can quantify the number of cells and/or the size of the metastatic foci would be important clinically.*

Reply:

As retraction of each sensing trap is an indication of the presence of at least one malignant cell in the CNB or FNA of the LN samples, the number of the involved traps could be an indication for a minimum number of detected metastasized cells. If you consider the conventional clinical H&E and Papanicolaou reports of the pathologists on the LNs of the patients (we presented four of them in below), they just state the involvement of the LNs by malignant cells or not in which they state "metastatic carcinoma" or "negative for malignancy". When more than one lymph node sample is assayed they mention to the number of involved samples e.g. 4/10 lymph samples are involved. Retraction of no sensing traps with respect to retraction of one or more sensing traps would be the boundary condition between presence or absence of metastasis in the sample assayed by MetasChip as expression of metastatic markers were

corroborated by IHC and RT-PCR from the other parts (that weren't assayed by MetasChip) of the same suspicious sample.

Patient ID:1, CNB of Breast and ALN

CLINICAL DATA:A spiculated mass in left breast 3 o'clock (BIRADs5)with suspecious lymph node
MACROSCOPIC:
Specimen received in two separate formalin containers labeled as:
1) Left breast mass: consist of six cores of gray- creamy, soft to rubbery tissue measuring 0.5to1.2cm in length and 0.2cm in diameter. Summary of specimen: 6/1 Embedded: 100%

2) Left Axillary lymph node: consist of three cores of gray- creamy, soft to rubbery tissue measuring 1to1.3cm in le and 0.2cm in diameter. Summary of specimen: 3/1 Embedded: 100%

MICROSCOPIC:
1) Sections show fragmented breast tissue infiltrated by malignant neoplastic tumor consists of nests of high N/C rati tumoral cells with pleomorphic large nuclei some with prominent nucleoli set in the sclerotic stroma. Some tumoral n surrounded by clefts are also seen
2) Sections show fragmented breast tissue infiltrated by malignant neoplastic tumor consists of nests of high N/C rati tumoral cells with pleomorphic large nuclei some with prominent nucleoli set in the sclerotic stroma.

DIAGNOSIS:
1) Left breast mass, 3o'clock, Core needle biopsy:
-Invasive ductal carcinoma with some area suspicious for micropapillary component .

2) Left Axillary lymph node, Core needle biopsy:
-Metastatic carcinoma
NOTE: For confirmation of micropapillary component IHC for MUC1 or EMA is recommended
ICDO: M: 8500.3
C: 50.9

Patient ID:2, CNB of Breast and ALN

CLINICAL DATA: 1)An irregular hypo echoic mass in left (BIRADs5)
2)Several hypo echoic lymph node in left breast
MACROSCOPIC:
Specimen received in two separate formalin containers labeled as:
1) Left breast mass 12 o'clock: consist of six cores of gray- creamy, soft to rubbery tissue measuring 0.3 to1cm. Summary of specimen: 6/1 Embedded: 100%

2) Left axillary lymph node: consist of four cores of gray- creamy, soft to rubbery tissue measuring0.5 to 1.5cm in length and 0.2cm in diameter. Summary of specimen: 4/1 Embedded: 100%

MICROSCOPIC:
1)Sections show fragmented breast tissue infiltrated by malignant neoplastic tumor consists of nests of high N/C ratio tumoral cells with pleomorphic large nuclei some with prominent nucleoli set in the severly inflamed stroma.
High mitotic figuers are also seen.
2) Section show lymph node architecture infiltrated by nest of tumoral cell with pleomorphic large nuclei
DIAGNOSIS:
1) Left breast mass 12 o'clock, Core needle biopsy:
-Invasive carcinoma with medullary future
-Micro calcification are also seen.

2) Left axillary lymph node, Core needle biopsy:
-Metastatic carcinoma

ICDO: M: 8510.3
C: 50.9

MEDICAL CYTOLOGY (OUTSIDE SAMPLE)

Specimen: FNA left axillary lymphnode.
Clin. data: Left breast mass.
See our, Path. Report. PLC96-667.

Received specimens: 10cc colorless fluid.

MICRO: E-Prep smears show:
-Numerous malignant tumor cells isolated and in clusters admixed with small lymphocytes.

CONCLUSION: FNA of left axillary lymphnode:
- **Positive for malignancy; compatible with metastatic carcinoma**.

MEDICAL CYTOLOGY (OUTSIDE SAMPLE)

Specimen: FNA of right axillary lymphnode.
Clin. data: Lymphnode with thick cortex in right axillary region.
NOTE: See our Path. Report No. PLC96-467.
Received specimens: 10cc colorless fluid.
MICRO: E-Prep smears show:
- Clusters of cohesive bland-looking epithelial cells (most probably from breast tissue).
- Very few lymphocytes in the background.

CONCLUSION:Designated as" FNA of right axillary lymphnode":
- **No cytologic evidence of malignancy**.

FigureR 7 – Pathological reports of patients ID 1,2(top) and ID 41,53(bottom)

Also please pay attention to the P-CK based IHC of the lymph node a patient(ID 38) whom wasn't diagnosed by H&E but positively scored by MetasChip (FigureR 8).More than 20 metastatic cells expressed P-CK were found. This reveal the importance of MetasChip in correcting the false results of H&E or Pap.

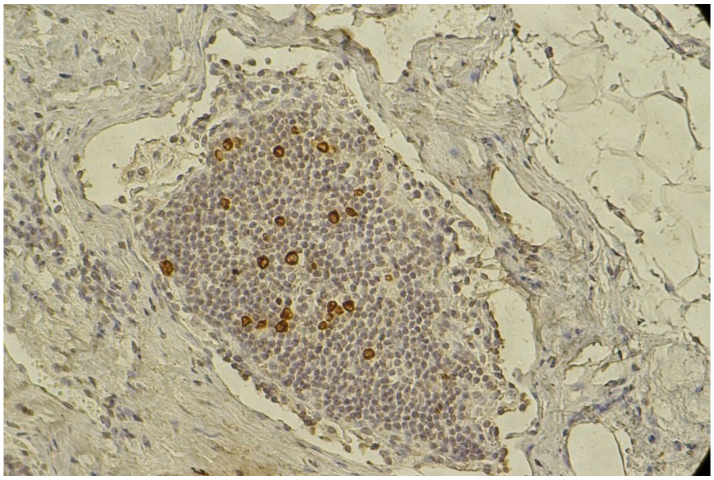

FigureR 8 - expression of P-CK in the lymph of patient ID:38, whom had been diagnosed as negative by H&E but positive by Metas-Chip. The sample presented more than 20 cancer cells expressed pancytokeratin

Moreover, the FNA studies added to the paper revealed the reliability of Metas-Chip as not only it diagnosed all Pap-malignant patients as positive, it detected the trace of metastatic cells in the lymph samples of 4 patients which were negatively scored in Pap staining and more in-depth IHC tests revealed positive EMA, PCK or CK-7 in their samples. As discussed earlier this also could shed new lights on the application of FNA in diagnosing the LN involvement because FNA were left and replaced by CNB due to its rare derived cells and non-reliable diagnosis. As FNA is less painful than CNB, metastatic diagnosis based on FNA in ALN might be achievable again with the assistance of Metas-Chip.

Reviewer #3 (Remarks to the Author):

Q: *I am extremely concerned that the manuscript has a very commercial feel to it and appears to over-simplify singular features that make a cell metastatic and an electrical characteristic that can be*

measured. My own experience with S100A8 research in the past 10 years has proven challenging to single out S100A8 as a predictive or therapeutic target (moreover, a number of the prognostic studies are underpowered.)

Reply

Thank you for your careful consideration. We only mentioned in introduction the role of S100 proteins in metastasis due to published papers in valid journals which investigated the mechanisms and reasons of cancer cells' tendency to metastasis [6, 8-10]. These references reported the role of chemokine based macromolecules in increasing the rate of cancer cell metastasis. However it could be true that the role of S100 chemokines in metastasis might be challenging (although we didn't found any publication which induce doubt about the role of the chemokine in metastasis, we modified the phrase in introduction due to the referee query).

Anyway, as this phrase wouldn't induce any problem in the result of our paper (because the tendency of cancer cells to invade the endothelial barrier is an established known phenomena apart from involved proteins [5, 6]. The important point is that we observed the filopodias with MMPs in their external region in metastatic cells as shown in actin and Anti pro MMP2 based confocal images and the videos (FigureR 6a). These results corroborate the attraction of malignant cells to attack and retract the HUVECs. Such interaction wasn't observed for non-malignant samples and immunoboold cells. (FigureR 6b,c, Supplementary Video S5 & S6).

Q: *Whereas the methodology appears to be reasonable, 20 patients is far too small a sample size in order to merit publication.*

Reply: Thank you for such a valuable query. We extended the assay to more than 70 patients with 20 more patients resected by CNB and 30 patients resected by FNA presented in Table1.

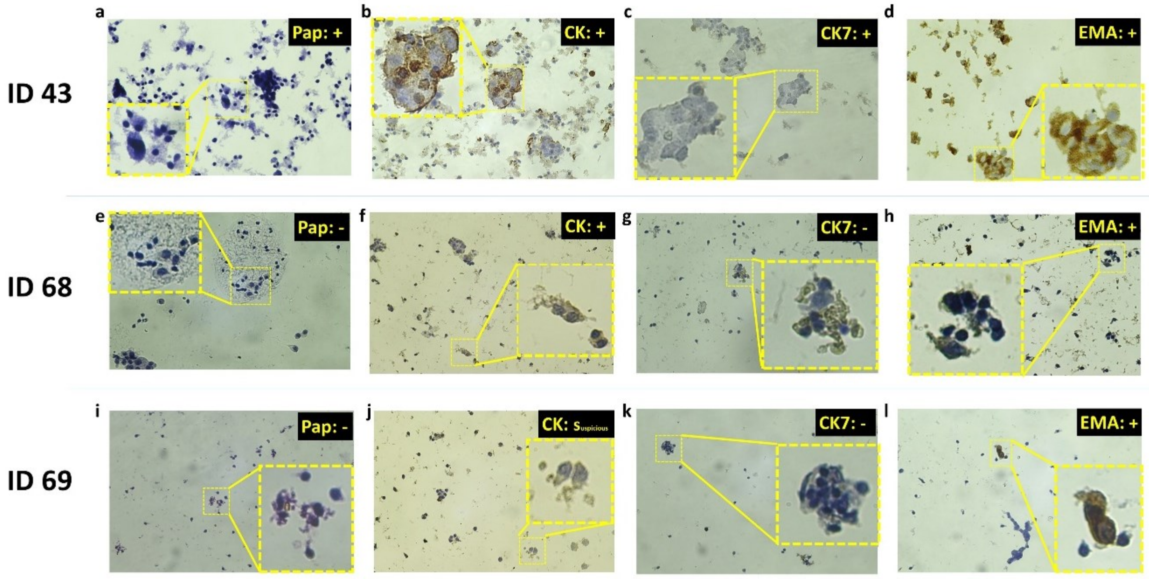

FigureR 9 –Cythopathological and immunohistochemical images of the lymph node aspirated from a known metastatic sample (ID 43) positively scored by MetasChip in comparison with the similar assays from two suspicious aspirated samples (ID 68 and 69) negatively scored in Pap stain but positively scored by MetasChip and IHC. (a) Cancer cells with large hyperchromic nucleus present metastatic carcinoma meanwhile no trace of malignant cells could be observed in (e & i). Expression of PCK in the cancer cells are observable in metastatic (b) and one of the suspicious (f) patient. Also the expression of PCK is suggestable in lymph of other suspicious patient (j). Expression of CK7 was positive in patient ID 43 (c) while it was negative in patient ID68 (g) and ID69 (k). Positive expression of EMA is observed in known metastatic (d) and suspicious patients (h & l). At least one IHC marker was positive in the suspicious patients who had been positively scored by MetasChip. Expression of metastatic epiflorescent marker (anti proMMP2) on malignant cells invaded HUVEC trap.

Similar to the assay on CNB samples, Metas-Chip detected the presence of metastatic cells in the FNA samples of the 16/30 patients (Table 1) in which the number of the assayed cells were much less than CNB samples.4/16 of the involved ALNs weren't diagnosed by conventional Pap staining method and the accuracy of Metas-Chip on those samples were corroborated by deep EMA, PCK and CK7 IHC investigations (FigureR 9). These are popular markers for tracing the ALN involvement in breast cancer [11] All of the FNA samples positively scored by Pap staining, had been diagnosed as involved ALNs in Metas-Chip(ID41-52).

The results were added to the paper.

Secondary comments:

Q: 1. *It would be helpful to know if the cells they captured would in fact produce metastasis in mice or if the cells are no longer viable after testing. If one could indeed show this in mice, one might have additional markers to look at (as the authors mentioned.)*

Reply: Injecting cancer cells into the mice is a complicated and non-reliable process, as we injected known 4T1 malignant breast cancer cell lines to 10 mices (FigureR 10) but the tumor just was formed in 4/10 of the mices. In vivo Tumorigenesis is a complicated process and other reports indicate that injecting a phenotypic transformed cell to other vital systems might not resulted in the same function [12, 13]. So, non-activated tumorigenesis in animal model would not be a reliable reason to exclude the malignant nature of the injected cells [12, 13].

Studies on diverse cancers, including melanoma, have indicated that only rare human cancer cells (0.1% to 0.0001%) have tumorigenic potential when transplanted into NOD/SCID mice [12]

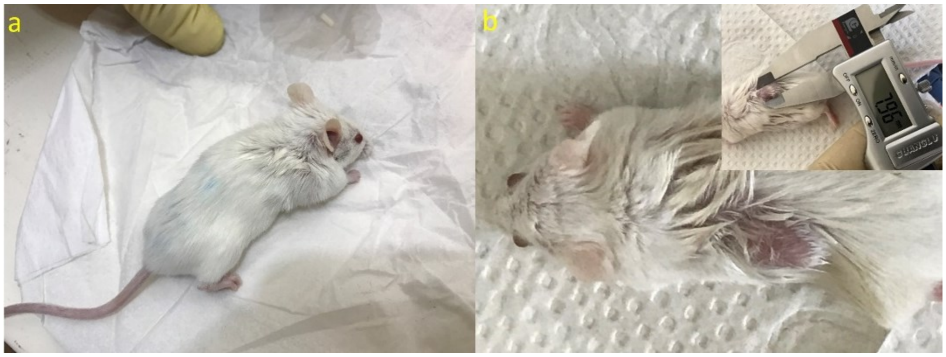

FigureR 10 - *Two mice were injected by similar concentration of 4T1 mice metastatic breast cancer cell line. A) Tumor wasn't formed even after 14 days b) Tumor was formed with a size of 7.96mm.*

Moreover we think that such injection to an animal could be replaced by confocal imaging from the invaded cells as (FigureR 6) showed the metastatic cells' filopodias which invasively entered the HUVECs and induced retraction and some signs of membrane blebbing in the HUVECs (FigureR 6a,Supplentary Video S7).

It is worth noting that all of the patients positively scored by MetasChip expressed at least one metastatic associated marker in their IHC assay and none of the samples negatively scored by MetasChip expressed any metastatic associated markers which could be traced in (Table 1) Moreover, pathological phenotype and membrane blebbing in HUVECs just could be induced by malignant cells as we discussed the non-perturbing interaction of non-malignant cells with endothelial layer in the reply of **referee#1 (Q1)**.

Reviewer #4 (Remarks to the Author):

The manuscript entitled "Metas-Chip identifies metastasis in biopsy samples of cancer patients faster 1 and more precise than conventional histopathological and immunohistochemical methods" is trying to demonstrate a microelectronic biochip (named Metas-Chip) that allows to detect the presence of metastasis in unprocessed samples. It was reported that the metastatic cells in the biopsied samples retract the traps's HUVEC, which induces sharp changes in electrical response. The metastasis in assayed breast cancer patients was identified with the accuracy of 100% using the Metas-Chip in less than 5 hours. In my opinion, the scientific merit and originality are enough to be published in this journal if the following concerns are addressed.

Reply:

Thank you very much for you interest and support on our work.

Major concerns

Q: 1. *More detailed information regarding biopsy preparation is required. For example, what size the biopsy should be? Where the biopsy should put in the biochip?*

Reply: The CNB specimens were in the range of 0.5 cm to 1.5 cm in length and about 0.3 cm in diameter. We remove less than 30% of each specimen for MetasChip assay. Subsequently we divide the sample to two or more separated parts and suspended them in DMEM media solution followed by pipetting for about 30 seconds. Then we put the solid sample (with reduced size because of pipetting of the solution) in, followed by injecting the peripheral solution (might be contain cluster or single suspicious cells) into

the reservoir. The image of the reservoir and a CNB sample before processing could be seen in Figure 1 of main article. In the case of FNA samples, which had been added in the revised edition, the sample already consists of suspended cells. The RBCs were removed from the sample with the assistance of Ficol followed by 20 minutes of centrifuge at 2000 RPMs. The RBC-removed sample was directly transferred to reservoir without any further processing. The reservoir of MetasChip fabricated by transparent bio-compatible PMMA could receive 500 uL sample.

The probable cancer cells in samples detach themselves and attack to HUVEC traps.

Q: 2. In Supplementary Figure 1, the effect of MCF7 on HUVEC was presented. However, more negative control experiments should be demonstrated besides MCF 7. In addition, please explain the number of experiments as showed in Supplementary Figure 1b

Reply: Thank you for such a valuable query. The MetasChip contains 6 pairs of electrodes which results in 6 individual sensing traps. We assayed MCF7 cell lines 5 times in separated assays. So 30 separated sensing traps were interacted by MCF7 cells and no retraction was observed. Moreover to exclude the role of non-malignant lymph cells such as lymphocytes and macrophages (due to your and referee 1 queries) we assayed the interaction of non-malignant lymph samples derived from Fine Needle Aspiration(FNA) of additional patients as well as WBCs of health blood donator with sensing traps of MetasChip. The results presented in FigureR 3.b showed neither retraction nor membrane blebbing of HUVECs. As a result no changes in electrical response was observed.

Finally we prepared non-malignant colon cell line HT-29. And assayed it by MetasChip similarly neither retraction of sensing traps nor electrical response signal were recorded by MetasChip

Q: 3. The manuscript claimed that “other types of the cells existed in the biopsied tissue like non-invasive epithelial cells, peripheral lipids and blood cells don’t apply invasive interaction by HUVEC traps so wouldn’t be captured by the Metas-Chip”. Although, the effect of lipid, WBCs and debris on HUVEC was showed in Supplementary figure 2, please explain the number of experiments as showed, as well as whether macrophages or immune cells caused the retraction of HUVEC.

Reply:

This is a valuable query. All of 23/70 assayed LNs, which weren’t malignant, didn’t apply any retractive and perturbing interaction with the sensing traps. To more clarify the non perturbing interaction between immunocells(such as macrophages and leukocytes) and HUVEC traps we presented a detailed comprehensive discussion with extensive experiments which could be observed below. Parts of the investigations were added to revised manuscript.

No invasive detachment or membrane blebbing (as an indication for cell death [3] of HUVEC sensing traps was observed in their interaction with non-cancerous lymph nodes or benign lymphadenopathies (which themselves contain a plenty of lymphocytes and macrophages because of inflammation induced by primary breast cancer)[4].

Due to your query and for more clarification a detailed comprehensive discussion was done based on both recently published nature review papers ([5]: *Nature Reviews Immunology ,2015; doi:10.1038/nri3908]: “How leukocytes cross the vascular endothelium”*)([6]: *Nature Reviews Cancer vo13 ,2013 , pp 858: “Crossing the endothelial barrier during metastasis”*) . Moreover we extended our

experiments on non-malignant lymph nodes and immune blood cells in interaction with HUVEC traps through cytopathological, confocal (FigureR 6) and time lapse imaging (FigureR 3). Which is added to the paper. Our findings exclude any immune cell (macrophages and lymphocytes)-HUVEC interaction that could trigger a false positive HUVEC trap response. Here we discuss our findings:

Leukocytes in turn instruct endothelial cells to open a path for transmigration. [5]

Danger signals such as inflammation stimulate resident cells of the innate immune system, such as mast cells, macrophages and dendritic cells, which leads to the secretion of cytokines and other pro-inflammatory mediators that activate nearby endothelial cells of the microvasculature. Thereby, a cascade of events is triggered that enables leukocytes to recognize the vascular endothelium and to interact with them through a series of steps known as capturing, rolling, leukocyte arrest, crawling to sites of exit and transmigration through the barriers of endothelial cells, pericytes and the basement membrane [5] (FigureR 11)

The important point is that with the assistance of LBRC and Actomyosin, the endothelial cells reseal themselves after extravasation of immune blood cells [5].

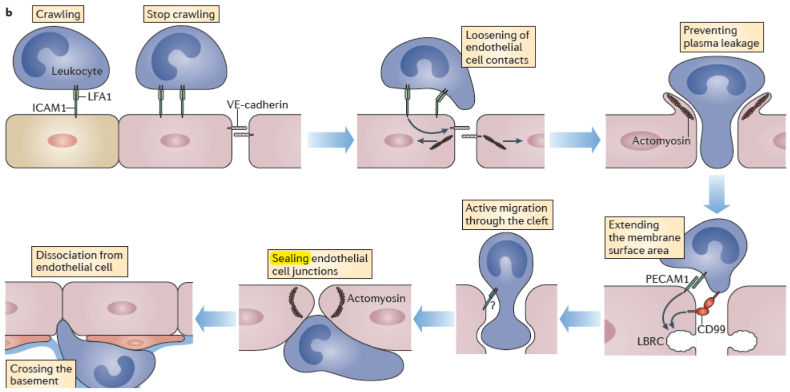

FigureR 11- The diapedesis process requires many functions mediated by leukocytes and endothelial cells: stopping intraluminal crawling at suitable exit sites; loosening of endothelial cell contacts; preventing plasma leakage; extending the membrane surface area at endothelial cell junctions through mobilization of the lateral border recycling compartment (LBRC); active leukocyte migration through the junctional cleft; and sealing of the junction after diapedesis. Finally, leukocytes dissociate from endothelial cells followed by transmigration through the basement membrane. CD99L2, CD99 antigen-like protein 2; ESAM, endothelial cell-selective adhesion molecule; ICAM, intercellular adhesion molecule; JAM, junctional adhesion molecule; LFA1, lymphocyte function-associated antigen 1; PECAM1, platelet endothelial cell adhesion molecule 1; VCAM1, vascular cell adhesion molecule 1. Secretion of such molecules indicated the pathway matching between HUVECs and vacation of blood cell[5].

Each of the markers and macromolecules have a known role in mediating the intra/extravasation of blood cells without perturbing or induction of membrane blebbing in the HUVECs. As an example:

CD99 was identified as a diapedesis-mediating receptor through the study of human CD99-specific antibodies that blocked monocyte migration through endothelial cell monolayers9. Similarly to PECAM1, CD99 was found on both monocytes and endothelial cells and was required on both cell types for diapedesis. Antibodies specific for mouse CD99 also inhibited lymphocyte entry into inflamed skin and neutrophil recruitment into the inflamed peritoneum [5]

The fact that the endothelial barrier can stay sealed despite the transmigration of leukocytes is remarkable, and it is likely that the endothelial actomyosin system is required to keep junctions tight while they enclose the diapedesing leukocyte. **So no pathological phenotypes or apoptotic/necrotic pathways would induce in endothelial cells during intra/extravasation of immune blood cells (**FigureR 12**)** [5].

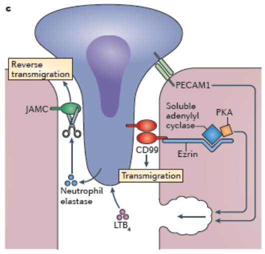

FigureR 12 - Diapedesing neutrophils trigger the mobilization of lateral border recycling compartment (LBRC) vesicles to the junctional plasma membrane of endothelial cells, which increases the membrane surface area at such sites. This effect is initiated by platelet endothelial cell adhesion molecule 1 (PECAM1) and CD99, with the latter signalling through the ezrin-located soluble adenylyl cyclase and protein kinase A (PKA). The leukocyte transmigration process is made irreversible by junctional adhesion molecule C (JAMC) Diapedesing neutrophils trigger the mobilization of lateral border recycling compartment (LBRC) vesicles to the junctional plasma membrane of endothelial cells, which increases the membrane surface area at such sites. This effect is initiated by platelet endothelial cell adhesion molecule 1 (PECAM1) and CD99, with the latter signalling through the ezrin-located soluble adenylyl cyclase and protein kinase A (PKA). The leukocyte transmigration process is made irreversible by junctional adhesion molecule C (JAMC) [5]

Movement of leukocytes through the endothelial cells may be supported by a multivesicular compartment, the lateral border recycling compartment (LBRC), inside endothelial cells. It was reported that this compartment is directly connected to the plasma membrane and readily mobilized to the cell

surface during leukocyte diapedesis, thereby helping to accommodate the body of the transmigrating leukocytes without entrance of endothelial cells to pathological phenotype [5].

Researcher findings reveal that the cancer cells secrete many proteins to perturb the HUVECs during extravasation [2, 6]. Also cancer cells trace many markers, secreted by HUVECs, to identify and invade the endothelial barriers [6].

Over the past few years, intravital imaging studies have revealed some of the mechanisms that underlie intravasation in vivo; for example, breast cancer cells in xenograft tumors have been shown to move directionally towards blood vessels[6].

Cancer cell extravasation usually occurs in small capillaries, where the cells can be physically trapped by size restriction and can then form stable attachments to endothelial. Meanwhile the leukocytes and macrophages just slide and transmit through endothelial cells by either paracellular or transcellular migrations [6].

A dynamic regulation of the endothelium by cancer cells through the formation of some membrane bridges was observed. (FigureR 6a)

The communication between the tumour cell and the endothelium upregulates markers associated with 'metastatic hijack', in which cancer cell-induced transformation of healthy endothelium into pathological endothelium and resulted in membrane blebbing and retraction of HUVECs sensing traps which was observed in time lapse and confocal images (FigureR 3, Supplementary Video S7). Such evidence wasn't observed in interaction non-malignant lymph nodes or WBCs with the traps (FigureR 3, Supplementary Video S5 & S6).

It is known cancer cells induce pathological phenotypes to all of the cells being invaded (such as vascular cells) during metastasis [2].

It has been reported that metastatic cells were found to preferentially form heterotypic connections to secondary tissues during metastasis [2],in our opinion, this might be the invadopodias we observed and showed by confocal (FigureR 6).

Neuronal cadherin (N-cadherin; also known as cadherin 2) is another receptor that is involved in the attachment and invasion of cancer cells [14].This receptor has no role in intra/extrvasation of imuno blood cells through endothelial barrier [4]. We showed over expression of N-Cadherin in malignant lymph nodes diagnosed by MetasChip meanwhile the expressing levels were low in safe lymph nodes (table..)

In summary, those references revealed that only cancer cells induce pathological perturbation into vascular barrier during invasion meanwhile the vascular cells can reseal themselves after intra/extravasation of non-cancerous immune blood cells. The reports indicated that transmigration of immune cells through vascular barrier is a preprogrammed process with known signaling pathways for endothelial cells [5]meanwhile invasion of cancer cells is a non-programmed process and a non-desired happening for vascular endothelial cells which induces pathological phenotypes in them. This was also seen *in vitro* by retraction and membrane blebbing of HUVEC traps in MetasChip. We observed such pathological transformation by membrane blebbing and retraction of HUVEC traps after being invaded by cancer cells (cell line and patients' samples)(FigureR 3c)(Supplementary Video S7) and those perturbations were absent for HUVECs being interacted by safe lymphs (Supplementary Video S5, FigureR 3a) and normal WBCs (Supplementary Video S6, FigureR 3b).

Giemsa cytopathological images also showed the HUVECs being perturbed by various MDAMB468 (FigureR 13).

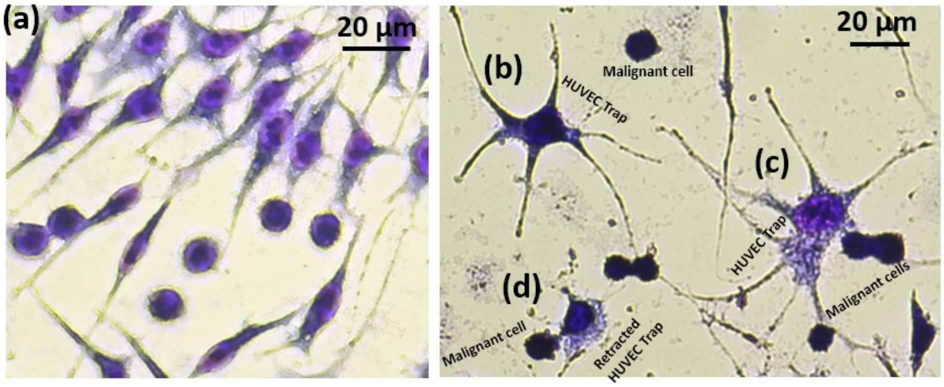

FigureR 13 - a) HUVEC traps after 7hr of interaction by Immuno blood cells b) Non invaded trap c) Start of invasion d) Invasion, start of retraction Perturbation of HUVECs due to invasion of metastatic cells were highlighted by additional confocal images

Similar images were taken from the interaction of non-malignant lymph nodes and HUVECs (fig a) no retraction was observed in the structure of interacted HUVECs.

Trace of transcellular and paracellular transmigrations of leukocytes during intra/extravasation through endothelial vascular layer without inducing any perturbation were reported (FigureR 14) [7]. We observed similar confocal images from non-retracted endothelial layer after TC and PC of blood immune cells(from non-metastatic patients) (FigureR 16.e-f)

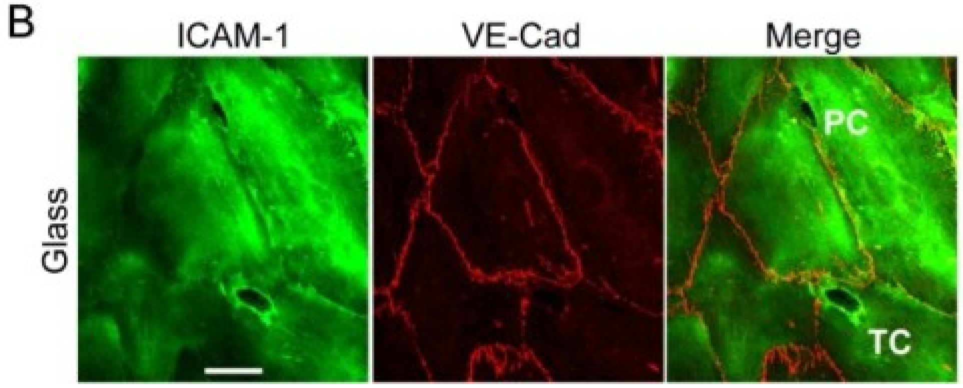

FigureR 15 - Representative confocal fluorescence images of cells taking the paracellular (PC) and transcellular (TC) routes. The migrating NK cells appear as small dark holes surrounded by intense anti-ICAM-1 staining, and the endothelial cell-cell junctions are visualized by anti-VE-cadherin staining. The endothelial cell substrate was glass in the upper panel and soft substrate (polyacrylamide) in the lower panel. Scale bar =10µm.[7]

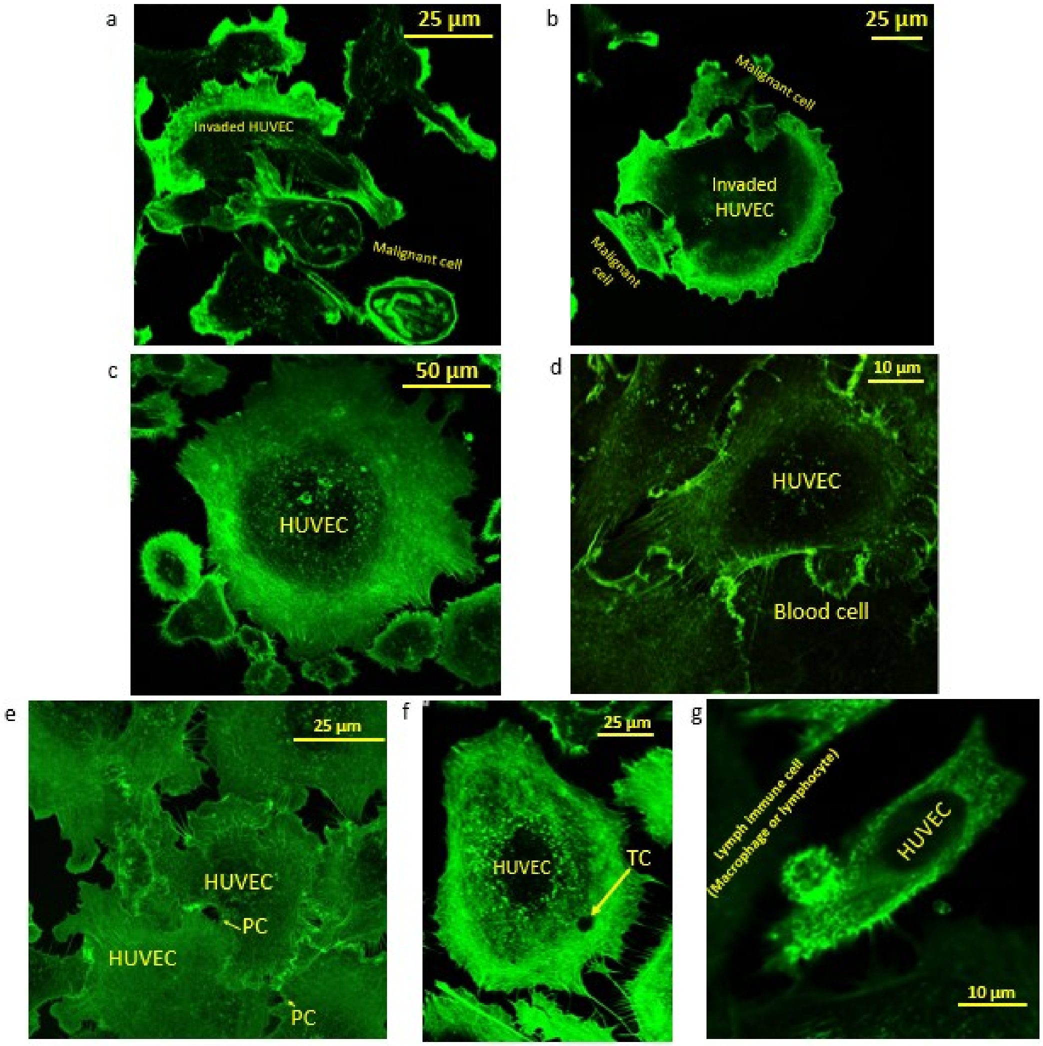

FigureR 16 - (a) Confocal images from interaction of two individual malignant cell samples by HUVEC traps. Retraction of the membrane and gins of membrane blebbing could be observed (b) Perturbation of HUVECs due to invasion of metastatic cells were highlighted by additional confocal images. (c) Similar images were taken from the interaction of non-malignant lymph nodes and HUVECs and no retraction was observed in the structure of interacted HUVECs. (e) The trace of the hole produced by transcellular migration of leukocytes could be observed. (f)Similar confocal images from non-retracted endothelial layer after TC of blood immune cells were reported by others. (g) Moreover attachment of an immuno cell existed in non-malignant lymph was observed but no retraction or membrane blebbing of the HUVECs was happened even after 6 hours.

Hence we observed no false positive result. The additional experiments on leukocytes and non-malignant lymph nodes were added to the paper.

Q 4. In figure 4, samples from 20 patients were analyzed. Their clinical information was needed. For example, what type of breast cancers are they? What stage? Whether are there correlation between the detection sensitivity and stage?

Reply: The number of assayed patients were increased to 70 prepared from both FNA and CNB samples. The clinical information about the charecteristics of thir breast cancer could be observed in TABLE SDA

Q:5. Besides breast cancers, the Metas-Chip could be applicable to other types of cancers, such as lung cancers, etc.

Reply: Thank you very much for such query. We extended our study on bone marrow aspirations in 4 pediatric cancers with the assistance of pediatric central Hospital to use MetasChip in diagnosis of bone marrow aspiration which is under progress (the results on 5 patients were so promising). Moreover the assay were carried on 2 Thyroid FNA to diagnose invasive thyroid cancer. Promising results were

achieved which we hope to continue them in future by extensive samples and complete corroborative immuno molecular and pathological assays. Also the potential application of MetasChip in detecting bladder cancer from urine and prostate cancer from biopsy sample are under progress.

Minor concerns

1. It is difficult to read the characters in figure.

Reply: we edited them

2. Rewrite the legend in Figure 1.

Reply: it has been rewritten.

1. Yilmaz, M. and G. Christofori, *EMT, the cytoskeleton, and cancer cell invasion.* Cancer Metastasis Rev, 2009. **28**(1-2): p. 15-33.
2. Connor, Y., et al., *Physical nanoscale conduit-mediated communication between tumour cells and the endothelium modulates endothelial phenotype.* Nature Communications, 2015. **6**: p. 8671.
3. Coleman, M.L., et al., *Membrane blebbing during apoptosis results from caspase-mediated activation of ROCK I.* Nat Cell Biol, 2001. **3**(4): p. 339-345.
4. Weiss, L.M. and D. O'Malley, *Benign lymphadenopathies.* Mod Pathol, 2013. **26**(S1): p. S88-S96.

5. Vestweber, D., *How leukocytes cross the vascular endothelium.* Nat Rev Immunol, 2015. **15**(11): p. 692-704.

6. Reymond, N., B.B. d'Agua, and A.J. Ridley, *Crossing the endothelial barrier during metastasis.* Nat Rev Cancer, 2013. **13**(12): p. 858-870.

7. Mukherjee, S., et al., *Role of Cortactin Homolog HS1 in Transendothelial Migration of Natural Killer Cells.* PLOS ONE, 2015. **10**(2): p. e0118153.

8. Rafii, S. and D. Lyden, *S100 chemokines mediate bookmarking of premetastatic niches.* Nat Cell Biol, 2006. **8**(12): p. 1321-1323.

9. Bresnick, A.R., D.J. Weber, and D.B. Zimmer, *S100 proteins in cancer.* Nat Rev Cancer, 2015. **15**(2): p. 96-109.

10. Stacer, A.C., et al., *Endothelial CXCR7 Regulates Breast Cancer Metastasis.* Oncogene, 2016. **35**(13): p. 1716-1724.

11. Niikura, H., et al., *Detection of micrometastases in the sentinel lymph nodes of patients with endometrial cancer.* Gynecol Oncol, 2007. **105**(3): p. 683-6.

12. Quintana, E., et al., *Efficient tumor formation by single human melanoma cells.* Nature, 2008. **456**(7222): p. 593-598.

13. Kim, J. and K.S. Zaret, *Reprogramming of human cancer cells to pluripotency for models of cancer progression.* The EMBO Journal, 2015. **34**(6): p. 739-747.

14. Nieman, M.T., et al., *N-Cadherin Promotes Motility in Human Breast Cancer Cells Regardless of Their E-Cadherin Expression.* The Journal of Cell Biology, 1999. **147**(3): p. 631-644.

Reviewer #2 (Remarks to the Author):

The authors have answered my questions, and have provided multiple additional data that strengthens the manuscript.

Reviewer #4 (Remarks to the Author):

All the concerns I raised are well addressed. However, the clinical issue is complex or diverse, and only a limited number of cases have been studied in this manuscript. I suggest, please make a discussion of the limitation or possible weakness in the section of discussion. In addition, please make change of the title with objective or precise phrase, if possible.

Reply to Referee#4:

Query: All the concerns I raised are well addressed. However, the clinical issue is complex or diverse, and only a limited number of cases have been studied in this manuscript. I suggest, please make a discussion of the limitation or possible weakness in the section of discussion. In addition, please make change of the title with objective or precise phrase, if possible.

Reply;

Thank you for your great considerations. We had experimented the Metas-Chip on more than 70 patients as presented in revised version. Moreover during these 70 days that our paper was under the consideration of referees we assayed 50 additive patients which is presented just in below because of the limited space of the paper for extensive results. Moreover, many of the papers published in nature journals presented the medical analyses on 20-50 patients [*Nature Methods 12, 685-691 (2015)*][*Nature Communications* **6**, 8671 (2015)]. Although we think it might be enough to validate the results, we still try to extend the number and types of the cases to present a system with broad applications in cancer diagnosis in early future.

In the case of your suggestion on adding a phrase about the weaknesses of the system, it is worth noting that as the Metas-Chip analyzes the samples in their live state. Although it would increase the precision, some observations such as starting the analysis maximum 3 hrs after removing the biopsy samples from the patients or maintaining the temperature of the sample reservoir before introducing to Metas-chip in 37 ^{o}C ought to be respected.

Moreover, some concerns such as quantitative grading of primary tumors and the threshold from micrometastasis to macrometastasis could be quantified based on the number and time interval of electrical spikes in sensing traps which are our future trends to enhance the efficiency of metals chip.

we add this phrase to the discussion section:

As the Metas-Chip analyzes the samples in their live state, some observations such as starting the analysis maximum 3 hr after removing the biopsy samples from the patients or maintaining the temperatire of the sample reserviour before introducing to Metas-chip in 37 ^{o}C ought to be respected.

Finally, some concerns such as quantitative grading of primary tumors and the threshold from micrometastasis to macrometastasis could be quantified based on the number and time interval of electrical spikes in sensing traps which are our future trends to enhance the efficiency of metals chip.

In the case of title, we edited the title to make it more precise and objective. Using from the biopsy samples in vital state, label-free mechanism of detection were highlighted and some additional phrases were removed:

"Metas-Chip precisely identifies presence of micro metastasis in live biopsy samples by label-free approach"

Finally I would like to thank all of the referees and editors for their useful and scientific comments which strongly improved the paper.
